# *De novo* design of insulated *cis*-regulatory elements based on deep learning-predicted fitness landscape

**DOI:** 10.1093/nar/gkaf611

**Published:** 2025-07-04

**Authors:** Haochen Wang, Yanhui Xiang, Ziming Liu, Wen Yin, Boyan Li, Long Qian, Xiaowo Wang, Chunbo Lou

**Affiliations:** Ministry of Education Key Laboratory of Bioinformatics; Center for Synthetic and Systems Biology; Bioinformatics Division, Beijing National Research Center for Information Science and Technology; Department of Automation, Tsinghua University, Beijing 100084, China; Center for Cell and Gene Circuit Design, State Key Laboratory of Quantitative Synthetic Biology, Shenzhen Institutes of Advanced Technology, Chinese Academy of Sciences, Shenzhen 518055, China; Center for Cell and Gene Circuit Design, State Key Laboratory of Quantitative Synthetic Biology, Shenzhen Institutes of Advanced Technology, Chinese Academy of Sciences, Shenzhen 518055, China; Center for Cell and Gene Circuit Design, State Key Laboratory of Quantitative Synthetic Biology, Shenzhen Institutes of Advanced Technology, Chinese Academy of Sciences, Shenzhen 518055, China; Center for Quantitative Biology, Academy for Advanced Interdisciplinary Studies, School of Physics, Peking University, Beijing 100871, China; Center for Quantitative Biology, Academy for Advanced Interdisciplinary Studies, School of Physics, Peking University, Beijing 100871, China; Ministry of Education Key Laboratory of Bioinformatics; Center for Synthetic and Systems Biology; Bioinformatics Division, Beijing National Research Center for Information Science and Technology; Department of Automation, Tsinghua University, Beijing 100084, China; Center for Cell and Gene Circuit Design, State Key Laboratory of Quantitative Synthetic Biology, Shenzhen Institutes of Advanced Technology, Chinese Academy of Sciences, Shenzhen 518055, China

## Abstract

Precise control of gene activity within a host cell is crucial in bioengineering applications. Despite significant advancements in *cis*-regulatory sequence activity prediction and reverse engineering, the context-dependent effects of host cellular environment have long been neglected, leading to ongoing challenges in accurately modeling regulatory processes. Here, we introduce an insulated design strategy to purify and model host-independent transcriptional activity. By integrating heterologous paired *cis*- and *trans*-regulatory modules into an orthogonal host cell, we established a controllable transcriptional regulatory system. Using a deep learning-based algorithm combined with an experimental data purification process, we achieved the *de novo* design full-length transcriptional promoter sequences driven by a host-independent activity landscape. Notably, this landscape accurately captured the transcriptional activity of the insulated system, enabling the generation of *cis-*regulatory sequences with desirable sequence and functional diversity for two distinct *trans*-RNA polymerases. Importantly, their activities are precisely predictable in both bacterial (*Escherichia coli*) and mammalian (Chinese hamster ovary) cell lines. We anticipated that *de novo* design strategy can be expanded to other complex *cis*-regulatory elements by integrating the deep learning-based algorithm with the construction of paired *cis*- and *trans*-regulatory modules in orthogonal host systems.

## Graphical Abstract

**Graphical Abstract:**
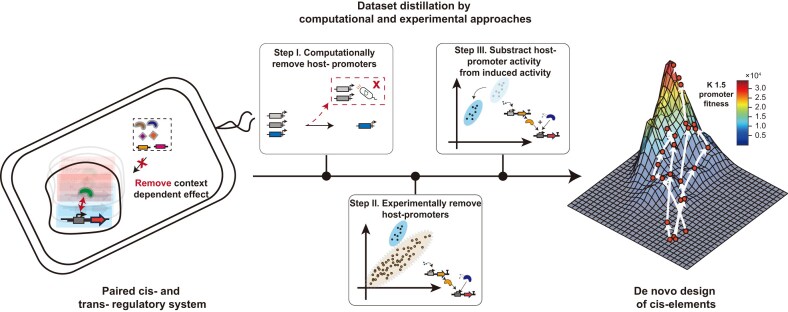

## Introduction

Biological information was encoded into the sequences of proteins, DNA, RNA, and other polymerized macromolecules [1–3]. Advances in deep learning algorithms have enable *de novo* structural predictions of transcription factors from their sequences [4–7]. However, predicting the transcriptional activity of these proteins remain challenging due to their complex interactions with *cis-*regulatory elements (e.g. promoter sequences, Transcription factor binding sites, etc.) [8, 9]. Meanwhile, these DNA- and RNA-elements, which were defined as *cis*-regulatory elements, were systematically investigated by combining high throughput experiments and deep learning (DL) techniques without varying their cognate *trans*-regulatory proteins in the host cells [10–14]. In fact, the expressions of the multiple *trans*-regulatory proteins would vary and interact with *cis*-regulatory elements of DNA and RNA to control gene expressions that shape the cell fate and fitness [9, 15].

The transcriptional activities depended on multiple *cis*-regulatory elements, such as core promoter sequences, transcription factor-binding sites, distal enhancers, DNA modification sequences, and nucleosome-disfavoring sequences [16–20]. Numerous DL algorithms were developed to predict transcriptional activities by varying one or more *cis*-regulatory elements [16,21–23]. However, most previous prediction models focused on the parts of region of the *cis*-regulatory sequences, but very few models tried to predict the full-length regulatory sequences from scratch. Additionally, most synthetic regulatory sequences were host-dependent and interacted in a complex manner with native *trans*-regulatory factors, which hindered the accurate prediction of transcriptional activity [24]. Previous attempts failed to construct the host-independent and self-explanatory regulatory system, preventing the full description and interpretation of *cis*-regulatory factors by deep learning or other computational approaches.

Here, we present a new insulated strategy for fully describing and *de novo* designing full-length promoter sequences based on the reconstruction of a paired *cis-*trans regulatory module in a heterogeneous host cell. The interaction between the *cis*-elements and the host native regulatory factors was disentangled by computationally and experimentally filtering out the host-dependent promoter sequences. Then, we constructed a DL-based framework for accurately predicting transcriptional activity and designing transcriptional regulatory sequences in a generative manner. With only a few samples to train the model, it has the potential to precisely predict the regulatory activity and depict the *cis*–*trans* insulated activity landscape of the system. Based on the landscape, we could successfully generate new regulatory sequences from scratch, and these sequences quantitatively kept their relative transcriptional activity when being transferred from bacterium to mammalian cells. Moreover, the insulated strategy was expanded to predict *cis*-regulatory sequences in other RNA polymerase (RNAP)-regulatory systems.

## Materials and methods

### The methods to remove host effect

**Figure 1.**
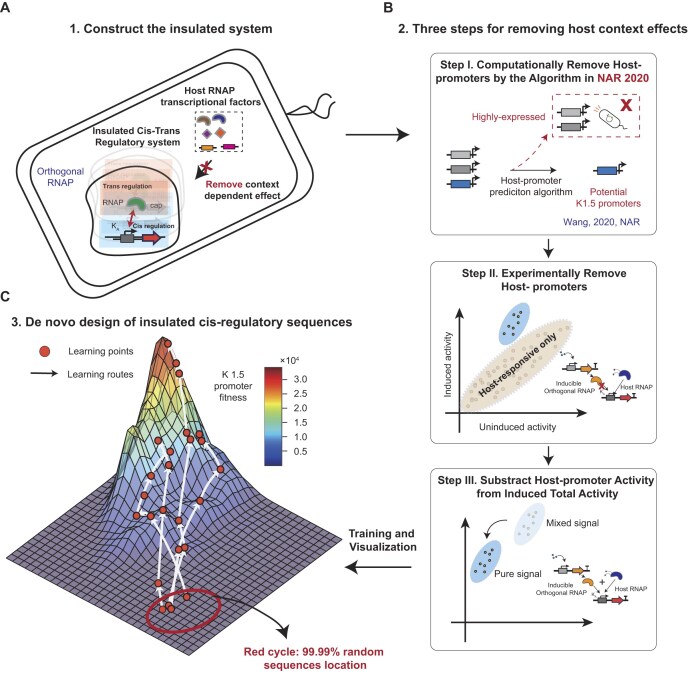
The overview of the insulated design strategy for *cis*–*trans* paired transcriptional system. (**A**) Scheme of the insulated *cis*–*trans* regulatory system by combining monomeric RNAP and its cognate promoters. (**B**) Three steps for purifying the training data pairs $( {\tilde s,T} )$ by both computational and experimental methods. (**C**) *De novo* design of promoter sequences from random sequences guided by the activity landscape which was learned by a CNN. The specific sequences are shown in Supplementary Table S3.

The major problem about K1.5 orthogonal promoter design is how to remove the *Escherichia coli* host-dependent context effects. We hypothesis that the final observation of gene expression is collectively contributed by the two factors, the K1.5 promoter system and the host-dependent promoter system. We first consider the characteristics of the K1.5 and *E. coli* as an example, we hypothesize that the K1.5 *cis*–*trans* system exhibits a less-rugged distribution in the sequence space, whereas the regulatory system derived from *E. coli* exhibits a rugged distribution (Fig. 1). Thus, in the sequence space we have:


\begin{eqnarray*}
{p_{ob}}\left( {T|s} \right) = {p_e}(T|s) \odot {\mathrm{\;}}{p_K}(T|s)
\end{eqnarray*}

\begin{eqnarray*}
{p_e}(T|s)\sim {\mathrm{\;}}{p_{{\mathrm{complex}}}}
\end{eqnarray*}

\begin{eqnarray*}
{p_K}(T|s)\sim {\mathrm{\;}}{p_{{\mathrm{simple}}}}
\end{eqnarray*}

where $s$ represents the sequence and $T$ represents the gene expression.${p_{ob}}( {T|s} )$ represents the observation distribution, ${p_e}(T|s)$ represents the *E. coli* distribution, and ${p_K}(T|s)$ represents K1.5 distribution. ${p_e}(T|s) \odot {\mathrm{\;}}{p_K}(T|s)$ represents the dependent sum of two distributions.

The problem is how to disentangle the ${p_K}(T|s)$ from the observation distribution ${p_{ob}}(T|s)$. Considering the characteristics of the K1.5 system, the first strategy we used is to ignore the *E. coli* host-dependent regions to simplify the problem. A prediction model was trained by *E. coli* gene expression data to remove the potential functional *E. coli* promoters. And experimental method was also used to filter the potential host-dependent sequences. To eliminate the influence of host-dependent expression levels, we also observed *E. coli* expression and removed the host-dependent expression ${T_e}{\mathrm{\;}}$from the observation expression ${T_{ob}}$. Thus, our goal is to estimate:


\begin{eqnarray*}
{p_K}\left( {T{\mathrm{|}}\tilde s} \right)\hbox{, where }T = {T_{ob}} - {T_e}.
\end{eqnarray*}

where $\tilde s$ represents sequence that nonfunctional in the *E. coli* system.

A convolutional neural network (CNN) was trained to estimate the distribution (Supplementary Fig. S8). Then we design functional sequence based on the distribution. The K1.5 promoter design problem can be formulated as maximizing the conditional probability ${p_K}( {\tilde s{\mathrm{|}}T} )$, according to the chain rules:


\begin{eqnarray*}
\mathop {\max }\limits_{\tilde s} {p_K}\left( {\tilde s{\mathrm{|}}T} \right) &=& \mathop {\max }\limits_{\tilde s} {p_K}\left( {\tilde s,T} \right)/p\left( T \right) \nonumber\\ &=& \mathop {{\mathrm{max}}}\limits_{\tilde s} {\mathrm{\;}}{p_K}\left( {T{\mathrm{|}}\tilde s} \right){\mathrm{*\;}}p\left( {\tilde s} \right)/{\mathrm{p}}\left( {\mathrm{T}} \right){\mathrm{\;}}
\end{eqnarray*}

Since property *T* is constant and determined by optimized goals, the objective of sequence design is proportion to:


\begin{eqnarray*}
\mathop {\max }\limits_{\tilde s} {p_K}\left( {\tilde s{\mathrm{|}}T} \right){\mathrm{\;}} \propto \mathop {\max }\limits_{\tilde s} {p_K}\left( {T{\mathrm{|}}\tilde s} \right){\mathrm{*}}p\left( {\tilde s} \right)
\end{eqnarray*}

The ${p_K}( {T{\mathrm{|}}\tilde s} )$ indicates the prediction model that trained to estimate the K1.5 distribution, and $p( {\tilde s} )$ restricts the optimization scope that only happen in the host-independent sequences.

According to the above theoretical results, we accomplish the three steps optimization work. In the first step, we got the training data pairs $( {\tilde s,T} )$ by both computational and experimental methods. In the second steps, we trained a CNN to estimate ${p_K}( {T{\mathrm{|}}\tilde s} )$, and visualize the functional landscape to verify our less rugged assumption. In the third steps, we design orthogonal promoters i.e. $\mathop {\max }\limits_{\tilde s} {p_K}( {\tilde s{\mathrm{|}}T} ){\mathrm{\;}}$ and test them in both *E. coli* and mammalian system. In addition, to thoroughly explore the orthogonal system, we analyzed the important motif that in the K1.5 promoter sequences.

### Bacterial strain and plasmids

Bacterial strains used in this study were *E. coli* K-12 DH10B and P_TAC_-T7 RNAP train, which was developed by integrating T7 RNA polymerase (T7 RNAP) and its Isopropyl β-D-1-thiogalactopyranoside (IPTG)-responsive regulatory elements into the genome of *E. coli* K-12 DH10B [25]. Plasmids used in bacterial work are from the backbone pPT or pRG [25]. Briefly, K1.5 RNAP was inserted into pRG vector by Gibson assembly. The resulting construct pRG-K1.5 RNAP makes the K1.5 RNAP expression under the control of IPTG. The construct was transformed into *E. coli* K-12 DH10B to generate strain K1.5 RNAP-DH10B.

For PK1.5wt, inserts K1.5 promoter sequence before RiboJ sequence in vector pPT by Gibson assembly. This resulting construct pPT-PK1.5wt makes the expression of reporter gene driven by K1.5 promoter. For K1.5 promoter random mutation libraries, the 21 bp core sequence in K1.5 wt promoter was mutated to N in the process of primer synthesis. Using the primer with N mutation, K1.5 promoter with random mutation was amplified by polymerase chain reaction (PCR). The PCR product was purified by Gel and PCR Clean Up Kit (Omega, D2000-2). The purified PCR product was then cloned into the plasmid pPT via Golden Gate Assembly and transformed into K1.5 RNAP-DH10B for the subsequent colony picking and *in vivo* analysis. The T7 promoter random library was generated following a protocol nearly identical to that used for constructing the K1.5 promoter random library. The key distinction lies in the T7 promoter library, which incorporates 1–8 base pair random mutations specifically within the T7 core promoter sequence. The backbone plasmid sequences were given Supplementary Tables S1 and S2.

For K1.5 promoter library, which was generated by deep learning, 60 000 oligos were synthesized by Nanjing GenScript Biotech Co., Ltd. The resulting oligos were amplified by PCR. Using the same strategy as K1.5 promoter random library, the deep learning library was generated and further analyzed.

Plasmids PK1.5wt and K.15 RNAP used in mammalian work are pOMC12 and pOMC7, respectively [26]. K1.5 promoter library plasmids were constructed by Gibson assembly using the primers with specific mutated K1.5 promoter sequence and pOMC12 as template.

### Random based K1.5 dependent promoter sequence selection

After transforming the random K1.5 promoter library into K1.5 RNAP-DH10B cells, the overnight cultured colonies on LBACI (LB Media with 100 μg/ml ampicillin, 25 μg/ml chloramphenicol, and 100 μM IPTG) plate were scraped using LBACI liquid media, take out one-third of the volume for cell sorting (BD, FACS Aria III). All the cells were divided into 10 bins depending on the fluorescence density. The colonies in the four strongest bins (>1000 colonies total) were plated on LBACI agar plates to separate single colony. After overnight culturing in 37°C incubators, put the plate under Blue LED transilluminator (Tiangen Biotech) to check the colony was fluorescent or not. The 96 colonies with fluorescence were transferred into LBAC liquid media with or without 100 mM IPTG in deep 96-well plate. After 14–16 h culture in micro-shaker, dilute the culture with Phosphate Buffered Saline (PBS) containing 1 mg/ml Kanamycin at the ratio of 1:200 in 96-well plate. Then, the fluorescence density was measured by flow cytometry (Beckman coulter Cytoflex S). Data were analyzed by CytoExpert Version 2.4.0.28. Meanwhile sequence the K1.5 promoters from colonies to identify mutations. Compare fluorescence intensities with and without IPTG: Promoters with unchanged fluorescence regardless of IPTG presence are considered host effect; Promoters with altered fluorescence in the presence of IPTG are identified as K1.5 RNAP dependent promoter. The procedure for selecting T7 RNAP-dependent promoters followed the same protocol as that described above for K1.5 RNAP.

### Cell line and transfection

Chinese hamster ovary (CHO) cells were cultured in high glucose Dulbecco’s modified Eagle’s media (DMEM-high glucose, Hyclone) containing 10% fetal bovine serum (FBS, GIBCO), and 1% penicillin–streptomycin (Hyclone). Cells were maintained at 37°C with 5% CO_2_. Cell lines were confirmed to be negative for mycoplasma during this study. Transfection procedure was followed by Lipofectamine 3000 transfection reagent (Invitrogen) standard protocol. Briefly, 24 h before transfection, plate 1.5 × 10^5^ CHO cells in each well of 24-well plate. When transfection, the cell confluency should reach up to 80%. For each well, 0.1 μg of pOMC12 or K1.5 mutated promoter plasmid and 0.9 μg of pOMC7 plasmid were diluted with 25 μl of DMEM-high glucose media in 1.5 ml centrifuge tube. After mixing the plasmids and DMEM media well, add 2 μl of p3000 reagent and re-mix well into the same tube. This is the master mix of DNA. Meanwhile, prepare lipofectamine 3000 mix in another 1.5 ml centrifuge tube: 2 μl of lipofectamine 3000 was diluted in 25 μl of DMEM-high glucose media. When both mixtures are ready, add master DNA mix to the diluted lipofectamine 3000mix. Mix well and incubate the DNA-p3000-lipofectamine mix at room temperature for 10 min. After that, add the mixture drop by drop onto the cells. Culture the transfected cells in a humidified incubator with 5% CO_2_ and 37°C for 24 h before flow cytometry analysis.

### The strength of K1.5 promoter qualification in bacteria and mammalian cells

The strength of K1.5 promoter was measured by flow cytometry. In bacteria, the single colony was cultured in Luria-Bertani (LB) liquid medium with antibiotics (LBAC) liquid media overnight. After that, dilute the overnight culture with M9ACI at ratio of 1:196 and continue culturing for 3 h. Dilute the 3 h culture with PBS containing 1 mg/ ml kanamycin at the ratio of 1:200 for flow cytometry analysis. Thirty thousand cells were collected for each sample. Data were analyzed by CytoExpert Version 2.4.0.28. The positive signal was gated by K1.5 RNAP-DH10B cells as negative control.

In mammalian cells, 24 h after co-transfection, cells were detached from the plate by trypsin with 0.25% EDTA (Gibco). After centrifuged at 1000 rpm for 5 min at room temperature, cells were resuspended in 4% paraformaldehyde solution (PFA) (Boster Biological Technology) and loaded onto flow cytometry (Beckman Coulter CytoFlex S). Hundred thousand cells were collected for each sample. Data were analyzed by CytoExpert Version 2.4.0.28. The positive signal was gated by the cells only transfected with pOMC12 plasmid as negative control.

### Experimental dataset for model training

The gene expression data, filtered using an *E. coli* host-dependent expression predictor, were cultured in a 96-well plate and analyzed through FACS. We selected 1252 sequences, each 31 base pairs long and free of mutation sites, to create our experimental dataset. In the K1.5 regulatory system, these sequences were tested in LB medium, with each experiment conducted in triplicate. The average gene expression from these three replicates was taken as the activity measurement for each regulatory sequence.

### Construct the gene expression prediction model

A CNN was employed to predict the gene expression of regulatory sequences. This network comprises four convolutional layers, two max pooling layers [27], and one fully connected layer, utilizing the “ReLU” activation function [28]. Each convolutional layer features a kernel length of five, and the kernel number in each layer is 100, 200, 200, and 5. For the training of the prediction model, the dataset was divided into 80% for training, 10% for validation, and 10% for testing. The mean squared error was selected as the loss function. The model was optimized using stochastic gradient descent [29], with a learning rate of 0.0005, a decay rate of 1 × 10^−6^, and a momentum of 0.9. We did not choose the hyperparameter values that resulted in the lowest error; instead, we used 95% of the dataset as the training set and then used the trained model to design novel sequences.

### Network structure

A CNN was trained to predict the gene expression from regulatory sequences. The structure of the network is listed as follows, and details could also be found in the github repo:

Predictor network structure:

- Input layer (dims = SeqLength * 4)
- Conv (Conv_kernelsize = 5, padding = 2, filters = 100)
- Activation_layer (ReLU)
- Conv (Conv_kernelsize = 5, padding = 2, filters = 200)
- Activation_layer (ReLU)
- Maxpooling(kernelsize = 2, padding = 0)
- Conv (Conv_kernelsize = 5, padding = 2, filters = 200)
- Activation_layer (ReLU)
- Conv (Conv_kernelsize = 5, padding = 2, filters = 5)
- Activation_layer (ReLU)
- Maxpooling(kernelsize = 2, padding = 0)
- Dense (units = 1)

We also tried CNN-LSTM and Attention-based neural network structures for comparison (Supplementary Figs S17 and S18, and Supplementary Table S7). The structure of the networks is listed below, and further details can be found in the GitHub repository.

- I. CNN-LSTM model
  - Input layer (dims = SeqLength * 4)
  - Conv2D (Conv_kernelsize = 5, padding = 2, filters = 100)
  - Activation_layer (ReLU)
  - MaxPooling (kernelsize = 2, padding = 0)
  - Reshape Layer (new shape = (SeqLength/2, 100))
  - LSTM (units = 200, return_sequences = True)
  - LSTM (units = 100, return_sequences = False)
  - Dense (units = 1024)
  - Dense (units = 1)
- II. Attention-based model
  - Input Layer (dims = SeqLength * 4)
  - Conv Layer (kernel size = 5, padding = 2, filters = 100)
  - Activation_Layer (ReLU)
  - MaxPooling (kernelsize = 2, padding = 0)
  - Reshape Layer (new shape = (SeqLength/2, 100))
  - Dense Layer (output_dim = 64) → Query vector/Key vector
  - Dense Layer (output_dim = 64) → Value vector
  - Attention Layer (scaled dot-product attention on query/key & value)
  - Flatten Layer
  - Dense Layer (units = 1024)
  - Dense Layer (units = 1)

### The semi-rational design procedure of regulatory sequences

Following the training of the CNN, we utilized it to identify regulatory sequences within specific expression ranges. Starting with sequences from our experimental dataset, we generated mutations at most eight sites difference from the original sequences (evaluated by edit distance). This process resulted in a mutated dataset of 10 000 sequences, from which we selected our target regulatory sequences. Our methodology achieved two main objectives:

- Range-based selection of regulatory sequences: We aimed to select sequences representing varying levels of gene expression activity compared to the most highly-expressed wild-type K1.5 regulatory sequence. These levels included fractions like 1, 1/2, 1/3, 1/4, 1/8, 1/16, 1/24, and 1/32 of the original expression activity.
- Diversity within fixed expression levels: The second goal was to choose sequences that not only exhibit similar gene expression activity but also maintain sufficient sequence diversity. Specifically, we selected 12 sequences, each demonstrating 1/16 of the gene expression activity of the wild-type K1.5 regulatory sequence.

This approach allowed us to systematically explore a wide spectrum of gene expression activities and sequence diversities within the context of the K1.5 regulatory system.

### The *de novo* design of regulatory sequences

Gradient-based optimization was employed to create regulatory sequences from random sequences. Assume our current input is an image $x$, and $J( x )$ is the function we seek to optimize (in our case, estimated using a neural network). The optimization process is carried out as follows:


\begin{eqnarray*}
{x_{new}} = {x_{old}} + \eta \cdot {\nabla _x}J\left( x \right)
\end{eqnarray*}

In this equation, we adjust the samples in the opposite direction of the gradient. Here, ${x_{new}}$ denotes the optimized sequence after one iteration, ${x_{old}}$ is the initial sequence, and $\eta$ represents the learning rate. For our study, we optimized the samples for up to 1000 iterations, setting the learning rate at 1. Ultimately, we selected regulatory sequences with activities of 5/4, 3/4, 1/2, 1/3, 1/4, 1/6, 1/8, 1/12, 1/16, 1/24, and 1/32 relative to the maximum gene expression of the wild-type K1.5 regulatory sequences.

### Draw the landscape of K1.5 regulatory system

The activity landscape in our study was visualized using feature maps derived from the penultimate layer of the neural network. Initially, experimental regulatory sequences were fed into the neural network, producing feature maps as outputs [30]. To construct the landscape, the maximum values from two distinct feature maps were utilized for the *x*-axis and *y*-axis, respectively. The *z*-axis was then formed based on the activities of the regulatory sequences. First, we extract the feature map from the last convolutional layer and select the outputs of two kernels as the two dimensions of the landscape. Next, the sequences used in the experiment are fed into the network, where the outputs of the two kernels are used as the two dimensions and the expression level as the third dimension to generate a 3D scatter plot. Finally, the scatter plot is used to construct the 3D landscape (Supplementary Fig. S16).

Three distinct types of landscapes were illustrated in our research:

- *E. coli* host-effect landscape: This landscape was generated using a neural network trained on data from uninduced gene expression. It represents the host-dependent activity of the regulatory sequences in the absence of specific induction stimuli.
- Orthogonal landscape: This was constructed using a neural network trained on the difference between induced and uninduced gene expression data. The orthogonal landscape highlights the activity obtained by the K1.5 system.
- Mixed landscape: In this landscape, we utilized a neural network that was trained on induced gene expression data. This landscape represents the activities of regulatory sequences under conditions influenced by both host and the K1.5 system.

### Draw the optimization route in the landscape

The optimization routes within the landscape were illustrated during the process of designing regulatory sequences from scratch. For each step of optimization, we employed the argmax function to identify the most likely regulatory sequence and then projected this sequence onto the landscape. To effectively visualize the optimization route for each sequence, we not only marked the start and end points on the landscape but also chose intermediate points. These points help to delineate the trajectory of the optimization process, offering the view of how the regulatory sequences evolve towards their optimized states within the given landscape.

### Neural network motif visualization

To extract the knowledge learned by each convolutional layer in the neural network, a motif visualization strategy is employed. The aim of this strategy is to identify the motif patterns learned by each convolutional kernel. The process consists of three key steps: (i) Feature Extraction: After training the activity prediction model and retaining the optimal weights, we input both natural promoter sequences and their mutant variants into the network to generate feature maps across a batch of sequences. (ii) Receptive Field Alignment: For each convolutional kernel, we locate the maximum activation positions in its feature map and map these positions back to the original nucleotide sequences based on the kernel’s predefined receptive field. (iii) Motif Construction: By aggregating all aligned subsequences within the receptive fields, we derive a Position-Specific Weight Matrix (PWM) representing the kernel’s learned motif. Finally, WebLogo is used to generate the WebLogo corresponding to the PWM. Further details have been provided in the “Materials and methods” section of the manuscript, and the detailed code is available at https://github.com/HaochenW/deepinsulated/tree/main/visualize.

We also added a t-SNE visualization of the first-layer convolutional kernels via dimensionality reduction (see Supplementary Fig. S15) and selected the most significant features from both the purified and nonpurified datasets. This selection was based on clustering using the *k*-means algorithm (performed in the original feature space, not the t-SNE-transformed space). Specifically, we used *k*-means clustering on the flatten of original PWM-derived feature vectors (i.e. prior to dimensionality reduction). For detailed algorithm, we selected the optimal number of clusters by computing the silhouette score for different values of *k* (ranging from 5 up to a maximum of 30). The number of clusters with the highest average silhouette score was chosen as the final *k*. To further highlight biologically meaningful patterns, we then identified “pure clusters”—clusters that contained only sequences from either the purified or the nonpurified dataset. A cluster was considered pure if: (i) the cluster contains at least three points of the same type grouped together; (ii) no points of the other type are mixed within the cluster; and (iii) the cluster is as far away as possible from other points.

### Gradient-based optimization

We utilized gradient-based optimization to optimize sequence activity. First, a trained model with determined weights was prepared as a guide for optimizing the sequences. Second, a group of sequences was generated purely randomly, and one-hot encoding was used to encode these random sequences. Third, these randomly encoded sequences were used as the starting point and were input into the neural network model to calculate the model’s gradient. Fourth, the sequences were optimized using the negative gradient direction. Starting from step two, this optimization process was repeated multiple times, allowing the sequences to gradually approach the high-expression region. Lastly, the base at each position was determined by selecting the position with the maximum value within the region. Note that there might be some cases that fall into the so-called “out of distribution” scenario, so the maximum value at each site was selected.

## Results

### Overview of the insulated *cis*- and *trans*-regulatory system construction

To construct heterologous paired *cis*- and *trans*-regulatory modules, we selected a monomeric RNAP from bacteriophage K1.5 [31] and its cognate promoter-reporter gene for building the insulated system (Fig. 1A). The initial host cell was chosen to be *E. coli*, after which our effort concentrated on obtaining a purified, high-quality dataset corresponding to the gene expression data from the system. The primary consideration was to remove the host cellular context-dependent effect, which is achieved in three steps (Fig. 1B).

In the first step, we computationally removed the *cis*-regulatory sequences with potential transcriptional activity in *E. coli* (see Fig. 1B, step I), because these sequences could introduce interference that complicates the modeling of the insulated system. Subsequently, we experimentally removed promoter sequences that did not respond to the heterologous RNAP, but only transcribed by the host RNAP (Fig. 1B, step II). Last, we experimentally isolated the pure transcriptional activity for the heterologous RNAP-responsive promoter sequences (Fig. 1B, step III). We got the data pairs $( {\tilde s,T} )$ in the step, where $\tilde s$ represents selected sequences from step I, and $T$ represents corresponding gene expressions obtained after step III.

After obtaining the high-quality dataset, our effort focused on developing a model capable of accurately depicting the system’s behaviors. To achieve this goal, we constructed a deep learning model to describe the gene regulation within the insulated regulatory system, revealing an insulated activity landscape (Fig. 1C, and Supplementary Fig. S1A and B). The model could establish a comprehensive connection between promoter sequences and gene expression activity, which can be depicted by:


\begin{eqnarray*}
{P_K}\left( {T{\mathrm{|}}\tilde s} \right)
\end{eqnarray*}

where ${P_K}$ represents the predictor.

Finally, guided by this landscape, we *de novo* designed regulatory sequences to build novel regulatory systems (Fig. 1C). The K1.5 promoter design problem can be formulated as maximizing the conditional probability ${P_K}( {\tilde s{\mathrm{|}}T} )$. In order to obtain target gene expression $T$, we have


\begin{eqnarray*}
\mathop {\max }\limits_{\tilde s} {p_K}\left( {\tilde s{\mathrm{|}}T} \right)\; \propto \mathop {\max }\limits_{\tilde s} {p_K}\left( {T{\mathrm{|}}\tilde s} \right)*p\left( {\tilde s} \right)
\end{eqnarray*}

The ${p_K}( {T{\mathrm{|}}\tilde s} )$ indicates the prediction model, and $p( {\tilde s} )$ restricts the optimization sequence scope that only occurs within the nonfunctional sequences in the *E. coli* system (see “The methods to remove host effect” in “Materials and methods” section). To achieve our goal, we developed two design (semi-rational design and *de novo* design) strategies, respectively, based on filtering methods and gradient-based optimization methods (Supplementary Fig. S2A and B). Consequently, we could design sequences from scratch to achieve specific gene expression levels based on the predictor algorithm.

### Random sequences were impracticable for generating orthogonal promoters for K1.5 RNAP

**Figure 2.**
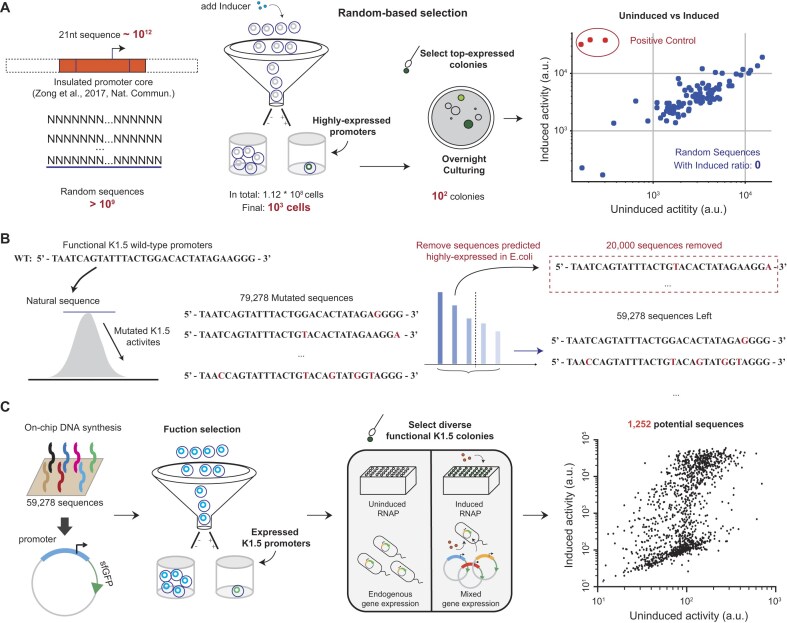
Design and construction of the library of *cis-*regulatory K1.5 promoter sequences. (**A**) Selection of the diverse and functional promoter library from random sequences. (**B**) Computationally remove the host-dependent promoter sequences derived from the wild-type K1.5 promoter sequence. (**C**) Oligo pool-based promoter synthesis and quantitative measurement of the functional K1.5 promoters.

The article by Zong *et al.*, published by our research group in 2017, discusses regions that play a regulatory role. We observed that for promoters based on the K1.5 and T7 systems, the regulatory sequences are highly localized and associated with specific regulatory proteins. This property is a key factor enabling the design of our insulation system. Based on insights obtained from *Zong et al*., we confirmed the insulated cognate promoter of K1.5 RNAP by constructing an IPTG-induced K1.5 RNAP expression (Fig. 2A and Supplementary Fig. S3). At the beginning, we tried to generate diverse functional K1.5 promoters by selecting active promoters from random sequences by flow cytometry (see “Random based K1.5 dependent promoter sequence selection” in “Materials and methods” section). We built an experimental framework to measure the reporter-gene expression activity of the system, using IPTG-induced activity to represent K1.5 RNAP-based promoter activity and IPTG-uninduced activity to represent the host RNAP-based promoter activity (Fig. 2A, see “Random based selection” in “Materials and methods” section). Considering that the sequence space of 21 bp sequences is 4^21^ (∼10^12^), we initially attempted to generate a huge number of random sequences in an effort to identify a subset of functional K1.5 sequences among them. Gene expression activities of >10^9^ random promoters (0.1% of the whole sequence space) were screened in IPTG-induced conditions and selected for diverse promoter sequences (Supplementary Fig. S3).

We hoped that selected sequences could be functional K1.5 promoters. Surprisingly, all randomly selected active promoters were not responsive to the IPTG-induced K1.5 RNAP expression (Fig. 2A, blue points). In contrast, the wild-type K1.5 promoters specifically responded to the K1.5 RNA expression (shown as red points in Fig. 2A), suggesting that these randomly selected active promoters responded to the host RNAP only. Based on the above observation, we presumed that the sequence space of functional K1.5 promoter sequences would be nearly negligible small region compared with the whole sequence space (Supplementary Fig. S4). Considering all the randomly selected functional sequences are host RNAP-dependent promoters for >10^9^ screened sequences, the host RNAP-dependent promoters would be much easier to be generated from a random sequence space than the K1.5 promoters. Thus, we needed computationally and experimentally removing the randomly emerged host RNAP-dependent promoters from the generated K1.5 promoter candidates.

### Screening for active K1.5 promoter candidates from a designed library

To obtain the functional K1.5 promoter library, we began by curating purified datasets (${\tilde {s}}, T$). At first, we hypothesized that the sequences surrounding the wild-type K1.5 promoter could easily generate the functional K1.5 RNAP-dependent promoters. We thus generated ∼80 000 mutated promoter candidates from the wild-type one (Fig. 2B and Supplementary fig. S5) and removed the sequences that contain high host (*E. coli*) RNAP-dependent activity based on a previous algorithm developed by Wang *et al.* (Supplementary Fig. S6) [32]. With the above steps, we obtained ∼60 000 sequences for oligo-pool synthesis and promoter-library construction (Fig. 2B and C).

Next, we experimentally measured the promoter activities of these sequences with and without the induced expression of K1.5 RNAP. To robustly acquire activity-diverse K1.5 promoter libraries, we first separated the synthesized promoter library into four sub-libraries (low, low-medium, medium, and high activity) by flow cytometry based on the fluorescent reporter gene expression (see “Flow cytometry and data analysis” in “Materials and methods” section). More than 1000 promoters were chosen from the four sub-libraries to create diverse promoter activities and were quantitatively measured and sequenced. We illustrated the relationship between uninduced and induced reporter-gene expression in Fig. 2C, observing that most promoters exhibited higher induced activities in comparison with the uninduced ones. Interestingly, there are still a subgroup of promoter candidates was not responsive to the K1.5 RNAP, but only responded to the host RNAP (Fig. 2C). We thus removed these host RNAP-responsive-only sequences from the training dataset for the deep learning model (Supplementary Fig. S7).

### Model accurately predicts orthogonal expression

**Figure 3.**
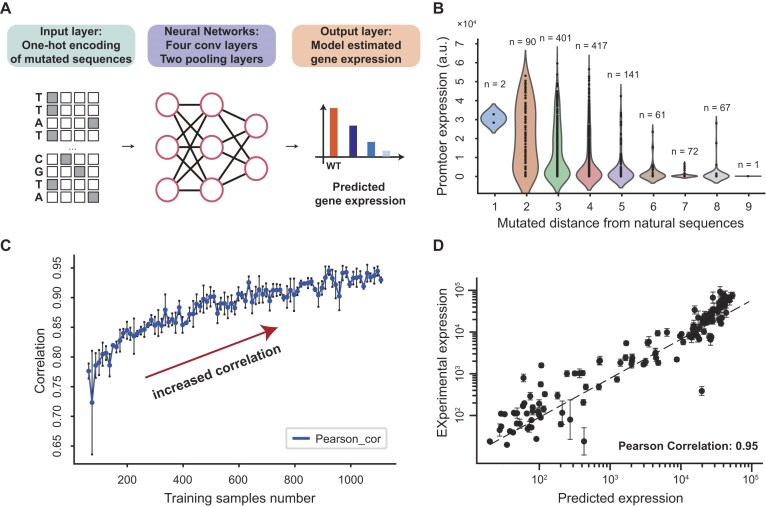
Training and testing the quantitatively measured K1.5 promoter library by the deep learning model. (**A**) Scheme of deep learning model to predict the activities of promoters. (**B**) The violin plot of gene expression with the increase of hamming distance to wild-type K1.5 promoter sequence. (**C**) The Pearson correlation between predicted and experimental promoter activity with the increase of training sample numbers. (**D**) The relationship between predicted and experimental promoter activity when using 90% samples for training and 10% samples for testing.

In the next steps, we attempted to construct the model to accurately depict the regulatory properties of the insulated system. CNNs [33], a type of DL models, have emerged as promising tools for predicting transcriptional regulation from regulatory sequences in recent studies. The network is constructed based on convolutional layer, which uses convolutional operator to capture the core motif features of transcription factor-binding sites [34–36]. In the previous section, we obtained a purified dataset of promoter sequences with diverse gene expression levels. Based on that, a CNN was constructed to capture the intricate features present in the promoter sequences (by using one-hot encoding) and predicted the gene expression within the K1.5 system (Fig. 3A, Supplementary Fig. S8, see “Construct the gene expression prediction model” in “Materials and methods” section). A total of 1252 promoters with sufficient sequence- and function-diversity were selected to form the training dataset (Fig. 3B and supplementary Data S1, see “Experimental dataset for model training” in “Materials and methods” section). As shown in Fig. 3B, gene expression decreases as the Hamming distance from the wild-type K1.5 promoter sequence increases (*t*-test, *P*-value < 0.05). We found that after model training, the square of Pearson correlation (*R*^2^) of testing set could reach 0.90 (Fig. 3C, *R* = 0.95, *R*^2^ = 0.90, Supplementary Data S2), which means >90% expression variance could be explained by the deep-learning model (Fig. 3D). Compared with CNN-LSTM, and attention-based models, CNNs have achieved comparable or even better results in our dataset (90% training set, 10% test set, 0.9510 ± 0.1 for CNN-based models, 0.9348 ± 0.1 for attention-based model, 0.8775 ± 0.2 for CNN-LSTM model). This is likely because the convolutional kernels can capture certain motif features and their combinatorial characteristics, which align closely with the promoter regulation patterns in bacteria, thus achieving good results. We then assessed whether this limited training dataset could reliably predict the promoter activity for K1.5 system. The DL models were trained with an increasing number of samples, and revealed that only a slight improvement in prediction accuracy when the sample size exceeded 300 (Fig. 3C), which suggests the size of our training set is enough to capture the dominance of gene regulatory properties. These results surpass those obtained using linear regression method, indicating the nonlinear interactions in the gene regulation could be well captured by our DL model (Supplementary Figs S9 and S10).

### Semi-rational design enables to generate defined-strength promoter sequences

**Figure 4.**
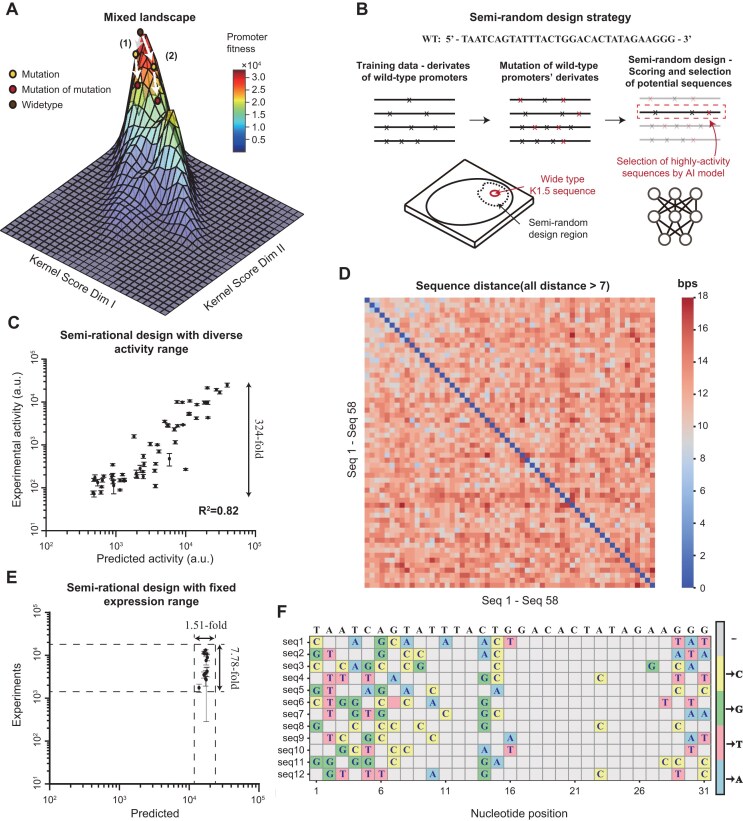
Semi-rational design of sequence- and functional-diverse insulated promoters derived from the wild-type K1.5 promoters. (**A**) The functional landscape of K1.5 promoter activity constructed by the last-layer kernel (kernel-1 and kernel-2) features. (**B**) Computational design of diverse sequences derived from the wild-type K1.5 promoter derivatives. (**C**) The experimental evaluation of the semi-rational designed promoter sequences with predefined activity (1, 2/3, 1/2, 1/3, 1/4, 1/8, 1/16, and 1/32 of the wild-type K1.5 promoter). (**D**) The sequence diversity of semi-rational designed promoters. (**E**) The experimental evaluation of the semi-rational designed promoter sequences with fixed activity. (**F**) The sequence mutations of model-designed samples in a fixed expression range corresponding to the wild-type K1.5 sequence, and its sequences are shown in Supplementary Table S4. Data represent the mean ± SD from at least three biological replicates.

One of the core demands of synthetic biology applications is to acquire sufficiently diverse sequence and functional *cis*-regulatory elements, in order to fine-tune gene expression and mitigate the risks of homologous recombination by ensuring sequence diversity. We constructed a landscape-guided design strategy to obtain sufficiently diverse sequence and activity mutated from wild-type ones. Initially, we recognized that a typical biological landscape is rugged and prevents the construction of their predictive models. However, we speculated that the activity landscape of the K1.5 promoter might not be heavily rugged, unlike the well-studied landscape of an enzyme with >500 peaks [37]. The underlying reason may be the interactions between the promoter and K1.5 RNAP were not extensive and complicated. We used weight matrices in the last layer of the trained DLs to construct an activity landscape of the K1.5 system, because the last layer combines all features to understand complex regulatory rules (Fig. 4A, see “Draw the landscape of K1.5 regulatory system” in “Materials and methods” section). We indeed found that the deep learning model could capture the activity landscape with several main peaks.

Based on this less-rugged activity landscape, a semi-rational design strategy was used to generate the diverse promoter sequences from the wild-type promoter (wt-promoter) (see “The semi-rational design procedure of regulatory sequences” in “Materials and methods” section), in which we deeply mutated the promoter-derivatives, acquired more diverse sequences and progressively searched for sequences that meet predefined expression levels by the above deep learning model (Fig. 4A and B). Obviously, this semi-rational design method cannot search the entire sequence space due to its focus on mutated sequences from the wild-type K1.5 promoter, but it was enough to generate the promoter sequences with desired activity and diversity. To generate diverse promoters with desired activity, we successfully designed 58 synthetic promoters with a predefined activity (1, 2/3, 1/2, 1/3, 1/4, 1/8, 1/16, and 1/32 of the wild-type K1.5 promoter). We then experimentally constructed the promoter-reporter gene cassette on a same plasmid backbone and measured their strength by flow cytometry (see “The strength of K1.5 promoter qualification in bacteria and mammalian cells” in “Materials and methods” section). The experimentally measured values were quite consistent with the predicted ones (squared Pearson correlation *R*^2^ = 0.82) (Fig. 4C and Supplementary Fig. S11), indicating that the semi-rationally designed promoters with gradient strength were precisely predicted (Fig. 4C). Besides functional diversity, these promoters exhibit sufficient sequence diversity with all sequences having at least 8 base-pair differences from each other (Fig. 4D), which is enough for avoiding homologous recombination [38]. Moreover, we also verified whether our model could accurately design diverse sequences for a fixed promoter strength. As shown in Fig. 4E, the measured strength of these designed promoters varied by 7.78-fold in comparison with the predicted promoters varied within 1.51-fold (Fig. 4E). Finally, we compared the hamming distances between the semi-rationally designed sequences and the wild-type K1.5 promoter sequence. Most sequences differed by >7 base pairs from each other, indicating their sequences are quite diverse (Fig. 4F and Supplementary Fig. S12). Together, all the tested functional- and sequence-diverse promoters supported the designability of the semi-rational design methods based on the wild-type promoter derivatives.

### Purification of experimental dataset enables to *de novo* design of functional K1.5 promoter sequence from scratch

**Figure 5.**
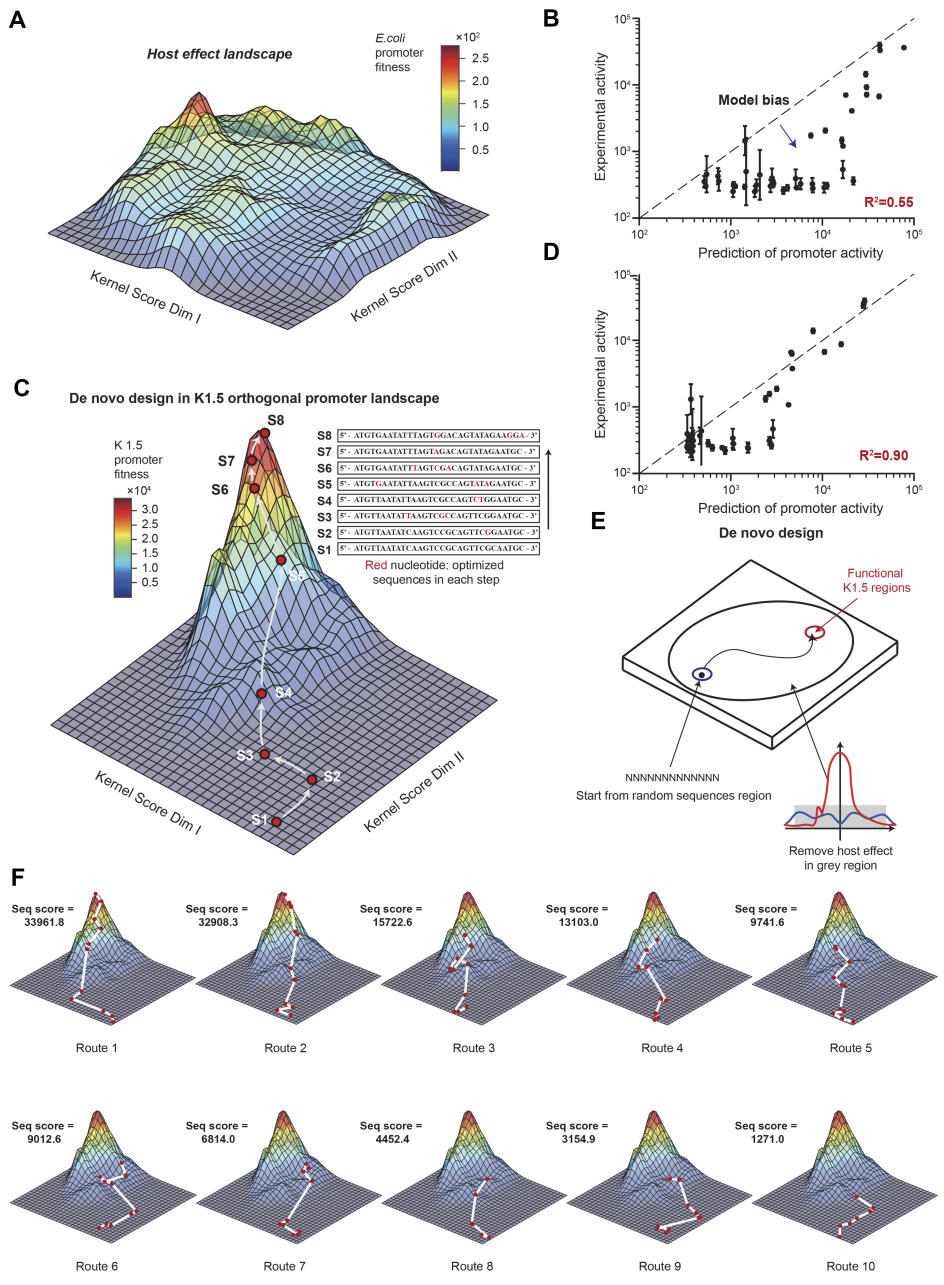
*De novo* design of sequence and functional-diverse insulated promoters from scratch. (**A**) The host effect landscape of *E. coli* is constructed by a trained neural network learned from uninduced promoter activity. (**B**) The experimental evaluation of the *de novo* designed promoter sequences with predefined activity. The bias between predicted and experimental results is mostly observed in promoters with low to medium activity. (**C**) The optimization trajectories of one *de novo* design highly activity promoter is shown in the landscape. Starting from completely random sequences, each red point represents a model-optimized sequence at each step of the optimization process. (**D**) The experimental evaluation of the *de novo* designed promoter sequences is based on a purified dataset with host-dependent activities subtracted. The bias between predicted and experimental results is mostly observed in promoters with low activity. (**E**) The scheme of *de novo* design strategy of functional promoters in K1.5 orthogonal promoter landscape. (**F**) Ten examples of optimization trajectories of *de novo* design of functional promoters in varied predefined activity. The sequence is optimized to demand activity through a variety of diversified routes. Data represent the mean ± SD from at least three biological replicates. The specific optimization sequences are shown in Supplementary Tables S5 and S6.

We next examined the generative design capability of the above semi-rational design model to create diverse functional K1.5 promoter sequences from scratch (Fig. 5). However, we found that most of the *de novo* designed promoter sequences were unpredictable and nonfunctional promoters except the high-activity promoters (Fig. 5B), indicating that the semi-rational design model could only capture the strong signals of the orthogonal promoters but not the moderate and weak ones. The possible reason is that the moderate- and low-activity were contributed by host-dependent effect in the semi-rational design model. We found that the host activity landscape was rugged and interrupted the optimization steps of functional promoter sequences (Fig. 5A).

We thus purified the experimental data by subtracting the host-dependent activity (uninduced activity) from the induced K1.5 promoter activity, and obtained a dataset of host-independent activity for training a new DL model (Supplementary Fig. S7). Based on the newly trained DL model and guided by the landscape with a gradient-based strategy, we *de novo* designed 41 promoter sequences started from random sequences (see “The *de novo* design of regulatory sequences” in “Materials and methods” section). To thoroughly investigate the optimization steps of gradient-based optimization, we draw the design route visualized in the orthogonal landscape (see “Draw the optimization route in the landscape” in “Materials and methods” section), showing the promoter sequences gradually improving from low-activity random sequence region to high-activity functional sequence region via multiple steps of gradient optimization (Fig. 5C). For all of 41 designed promoter sequences, surprisingly, we found that the predictability of the promoter sequences was dramatically improved (squared Pearson correlation increased from *R*^2^ = 0.55 to *R*^2^ = 0.9) when host-dependent activities are subtracted from the induced promoter activity (Fig. 5D). Simultaneously, we observed several very low-activity promoters were still hard to be predicted, possibly due to influence of the noisy signals of the fluorescent reporter. Interestingly, when mapping their *de novo* design paths, we observed that all of them started from the random-sequence region and gradually climbed towards different endpoints (Fig. 5E and F). With each step, the algorithm optimized 1∼3 base pairs to improve the transcriptional activity. The sequences started from completely different random sequences, and they were significantly different from each other (Supplementary Fig. S13 and Supplementary Data S3). Furthermore, we calculated the distance between designed sequences and wild-type sequences, and found that *de novo* designed sequences show higher sequence diversity than semi-rational design strategy. Besides, the sequences generated by *de novo* design are mostly >10 base pairs distant from the wild-type K1.5 sequence, indicating more sequence diversity compared with semi-rational design (Supplementary Fig. S14).

### The model interpreted the orthogonal *cis*-regulatory features

The deep learning model trained on the purified dataset achieved significantly better prediction results and successfully designed orthogonal promoters from scratch. Thus, we are curious about the reasons behind the increased orthogonality in the model. To explore the origins of this orthogonality, we employed network interpretation methods to compare the models trained with and without the purified experimental dataset. The interpretation of convolutional kernels was accomplished using the maximal activation strategy, which involves identifying sequence patterns that elicit the maximum activation for each kernel. The features represented by the convolutional kernels progressively became longer with increasing layers and learned motifs from different layers of a CNN were extracted, representing features at various scales (Supplementary Fig. S15A and B). Many motifs exhibit similar patterns between models trained on purified and nonpurified datasets; for example, motif logo 3 in the purified model corresponds to motif logo 7 in the nonpurified model. However, there are also significant differences, such as motif logo 1, which differs between the purified and nonpurified models.

To identify orthogonal features unique to the purified model, we employed t-SNE to visualize the differences in motif features between the two models. The t-SNE results help us identify four important orthogonal features in the neural networks (Supplementary Fig. 15 and Supplementary Table S7). One of the motifs located at position 23 represents the TATA-binding motifs of the promoter.

### *De novo* design enables to generate cross-species orthogonal regulatory sequences

**Figure 6.**
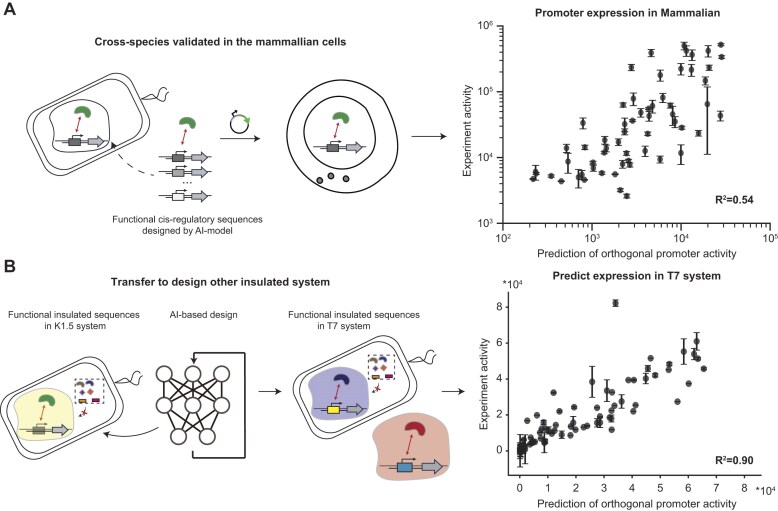
Generalizability of the performance of the designed K1.5 promoters in mammalian cells and insulated design strategy on other simple orthogonal systems. (**A**) Cross-species validation of model-designed K1.5 insulated promoter activity in mammalian cells. (**B**) Transferring insulated design strategy into T7 promoter-RNAP paired regulatory system. The figure on the right illustrates relationship between the predicted and experimental validated the predictability of insulated promoter activity in T7 orthogonal system by the DL models. Data represent the mean ± SD from at least three biological replicates.

To assess the cross-species ability of the designed K1.5 promoters, we measured their activity in a mammalian (CHO) cell line. Following our previous work [25], we reconstructed the RNAP and promoter pairs in a mammalian plasmid backbone, and a capping gene was fused to the N-terminal of the RNAP gene to create the mature capped mRNAs for reporter-protein translation. The fluorescent reporter was quantitatively measured by flow cytometry. The results show that the measured activity in mammalian cells maintained a linear relationship with the predicted activity in bacteria (Fig. 6A, *R*^2^ = 0.54), indicating that the orthogonal promoter activity can be quantitatively maintained when moving from bacteria to mammalian cells.

The success of the designed K1.5 promoters encouraged us to expand the insulated design strategy to other regulatory sequences. T7 promoters, known as highly efficient *in vitro* and *in vivo* transcription system, were chosen as an example to examine the predictive ability of our insulated design strategy. We found that their activity was precisely predicted (Fig. 6B). This shows that our method is flexible and can be used to create desired promoters in other regulatory systems.

## Discussion

In this study, we initially proposed an insulated design strategy for constructing a *cis*- and *trans*-regulatory system in a heterogeneous host cell. Recently, AI-based design approaches had achieved significant advancements in the *cis*-regulatory sequence design. However, a major challenge remains: *cis*-regulatory elements are inherently interacted with numerous host factors, including regulatory RNAs, RNAPs, transcription factors, epigenetic modifications, and chromosomal three-dimensional structure [39–41]. Current designs often focus solely on the element sequences themselves and ignore the change of the host factors, resulted in host- and context-dependent prediction and hard to be used in other contexts and other species. We thus reconstructed the *cis*- and *trans*-paired regulatory system in a heterogeneous host cell, then computationally and experimentally removed the host and context effect on the activity of the regulatory elements, and finally acquired high-quality purified dataset for training the CNNs and other deep learning models. We also confirmed the cross-species capability of the orthogonal designed *cis*-regulatory elements from bacterium to mammalian cells.

In recent years, regulatory sequence design has advanced rapidly. We and others have developed a range of methods for promoter design. Wang *et al.* developed deep generative models for promoter design [32]. Similarly, Zhang *et al.* designed the flanking sequences of promoters, successfully achieving the design of inducible promoters [21]. Boer *et al.* used machine learning models trained on entirely random sequences in yeast, generating yeast promoters capable of achieving specific expression levels in targeted cells [11]. However, all these methods share a significant limitation: they do not decouple the regulatory system from the host cell. The widespread presence of host-dependent regulation causes these promoters to function only in certain cells, and even perturbations in the intracellular environment can lead to variations in outcomes. In our work, we advance beyond the aforementioned approaches by separating endogenous signals from exogenous signals. We designed a regulatory system independent of endogenous expression and developed *de novo* sequence designs to achieve this. A deep learning-based algorithm combined with experimental data purification process enabled us to *de novo* design full-length transcriptional promoter sequences based on host-independent activity landscape.

Utilizing a deep learning framework and a purified dataset, we accurately predict transcriptional activity and design completely new promoters from random sequences. However, we still noticed that the low-activity (<5-fold of the background fluorescence) promoters were failed to be predicted, possibly due to the high background signal noises of the fluorescent reporter system. A more sensitive reporter, such as luciferase, would increase the predictability of the promoter sequences within the low-activity range.

As a simplest *cis*-*trans* regulatory pair, K1.5 promoter and cognate monomeric RNAP was chosen to demonstrate the insulated design strategy. One of the reasons why the number of training samples is much smaller than the number of free parameters is that neural networks can effectively reuse features through weight sharing and redundancy, which reduces the effective number of parameters. Additionally, for more complex systems that involve intensive interactions, a larger number of training samples would be necessary to achieve reliable model performance. For example, in the work by Boer *et al.* [11], they achieved a similar Pearson correlation using nearly 100 million random promoter sequences.

We first observed that most of random sequences generally did not contain K1.5 promoter activity, but was the host RNAP-dependent promoters. Therefore, derivates of wild-type K1.5 promoter were used to generate diverse functional K1.5 promoter library. We found that this DL model was sufficient to precisely predict transcriptional strengths trained on a relatively small dataset (∼1000 samples in 10^12^ sequence-space). Though the system is simple, it is important to note that this is just the beginning of the exploration. Determining what kind of data can cover what level of complexity and how to extend this to more complex systems are challenges we face in the near future. For instance, the number of combinations of *cis*- and *trans*-regulatory elements increase, the system's complexity would grow exponentially, and how much data required for predicting the complex systems would be a challenging problem too.

## Supplementary Material

gkaf611_Supplemental_Files

## Data Availability

Supplementary Data is available at NAR online. The computer source code is available from the public GitHub repository (https://github.com/HaochenW/deepinsulated) and from Zenodo (https://doi.org/10.5281/zenodo.15585877).

## Supplementary data

Supplementary data is available at NAR online.
