# Supplementary material for: De novo design of insulated cis-regulatory elements based on deep learning-predicted fitness landscape
Source: Nucleic Acids Res. 2025 Jul 4;53(12):gkaf611. doi: 10.1093/nar/gkaf611 (PMC12231593; doi:10.1093/nar/gkaf611)
Supplement: gkaf611_Supplemental_Files [file gkaf611_supplemental_files.zip › Supplementary Information_clean.pdf]

Supplementary information

***De novo* design of insulated transcriptional regulatory system based on deep learning-predicted landscape**

In the format provided by the authors and unedited

Supplementary Tables

Table S1 Sequences of Crucial Parts of the K1.5 promoter reporter and K1.5 RNAP Backbones in *E.Coli* DH10B

Name	Sequence
Terminator	cgtgccgaagaaaggcccacccgtgaaggtgagccagtgagttgattgctacgcgttt
Golden Gate marker	TGGTGAAGAGCctagagcaatacgcaaaccgcctctccccgcgcgttggccgattcattaatgcagctggcacgacaggtttcccgactggaaagcgggcagtgagcgcaacgcaattaatgtgagttagctcactcattaggcaccccaggctttacactttatgcttccggctcgtatgttgtgtggaattgtgagcggataacaatttcacacatactagagaaagaggagaaatactagatggcttcctccgaagacgttatcaaagagttcatgcgtttcaaagttcgtatggaaggttccgttaacggtcacgagttcgaaatcgaaggtgaaggtgaaggtcgtccgtacgaaggtacccagaccgctaaactgaaagttaccaaaggtggtccgctgccgttcgcttgggacatcctgtccccgcagttccagtacggttccaaagcttacgttaaacacccggctgacatcccggactacctgaaactgtccttcccggaaggtttcaaatgggaacgtgttatgaacttcgaagacggtggtgttgttaccgttacccaggactcctccctgcaagacggtgagttcatctacaaagttaaactgcgtggtaccaacttcccgtccgacggtccggttatgcagaaaaaaaccatgggttgggaagcttccaccgaacgtatgtacccggaagacggtgctctgaaaggtgaaatcaaaatgcgtctgaaactgaaagacggtggtcactacgacgctgaagttaaaaccacctacatggctaaaaaaccggttcagctgccgggtgcttacaaaaccgacatcaaactggacatcacctcccacaacgaagactacaccatcgttgaacagtacgaacgtgctgaaggtcgtcactccaccggtgcttaataacgctgatagtgctagtgtagatcgctactagagccaggcatcaaataaaacgaaaggctcagtcgaaagactgggcctttcgttttatctgttgtttgtcggtgaacgctctctactagagtcacactggctcaccttcgggtgggcctttctgcgtGCTCTTCA
Ribozyme-based insulator	AGCTGTCACCGGATGTGCTTTCCGGTCTGATGAGTCCGTGAGGACGAAACAGCCTCTACAAATAATTTTGT
sfGFP (reporter gene)	ATGCGTAAAGGCGAGGAGCTGTTCACTGGTGTCGTCCCTATTCTGGTGGAACTGGATGGTGATGTCAACGGTCATAAGTTTTCCGTGCGTGGCGAGGGTGAAGGTGACGCAACTAATGGTAAACTGACGCTGAAGTTCATCTGTACTACTGGTAAACTGCCGGTACCTTGGCCGACTCTGGTAACGACGCTGACTTATGGTGTTCAGTGCTTTGCTCGTTATCCGGACCATATGAAGCAGCATGACTTCTTCAAGTCCGCCATGCCGGAAGGCTATGTGCAGGAACGCACGATTTCCTTTAAGGATGACGGCACGTACAAAACGCGTGCGGAAGTGAAATTTGAAGGCGATACCCTGGTAAACCGCATTGAGCTGAAAGGCATTGACTTTAAAGAAGACGGCAATATCCTGGGCCATAAGCTGGAATACAATTTTAACAGCCACAATGTTTACATCACCGCCGATAAACAAAAAAATGGCATTAAAGCGAATTTTAAAATTCGCCACAACGTGGAGGATGGCAGCGTGCAGCTGGCTGATCACTACCAGCAAAACACTCCAATCGGTGATGGTCCTG

	TTCTGCTGCCAGACAATCACTATCTGAGCACGCAAAGCGTTCTGTCTAAAGATCCGAACGAGAAACGCGATCATATGGTTCTGCTGGAGTTCGTAACCGCAGCGGGCATCACGCATGGTATGGATGAACTGTACAAATGA
Composite terminator	TGATAAGCCAGGCATCAAATAAAACGAAAGGCTCAGTCGAAAGACTGGGCCTTTCGTTTTATCTGTTGTTTGTCGGTGAACGCTCTCTACTAGAGTCACACTGGCTCACCTTCGGGTGGGCCTTTCTGCGTTTAT
Constitutively expressed lacI (in reverse orientation)	TCACTGCCCGCTTTCCAGTCGGGAAACCTGTCGTGCCAGCTGCATTAATGAATCGGCCAACGCGCGGGGAGAGGCGGTTTGCGTATTGGGCGCCAGGGTGGTTTTTCTTTTCACCAGTGAGACTGGCAACAGCTGATTGCCCTTCACCGCCTGGCCCTGAGAGAGTTGCAGCAAGCGGTCCACGCTGGTTTGCCCCAGCAGGCGAAAATCCTGTTTGATGGTGGTTAACGGCGGGATATAACATGAGCTATCTTCGGTATCGTCGTATCCCACTACCGAGATATCCGCACCAACGCGCAGCCCGGACTCGGTAATGGCGCGCATTGCGCCCAGCGCCATCTGATCGTTGGCAACCAGCATCGCAGTGGGAACGATGCCCTCATTCAGCATTTGCATGGTTTGTTGAAAACCGGACATGGCACTCCAGTCGCCTTCCCGTTCCGCTATCGGCTGAATTTGATTGCGAGTGAGATATTTATGCCAGCCAGCCAGACGCAGACGCGCCGAGACAGAACTTAATGGGCCCGCTAACAGCGCGATTTGCTGGTGACCCAATGCGACCAGATGCTCCACGCCCAGTCGCGTACCGTCCTCATGGGAGAAAATAATACTGTTGATGGGTGTCTGGTCAGAGACATCAAGAAATAACGCCGGAACATTAGTGCAGGCAGCTTCCACAGCAATGGCATCCTGGTCATCCAGCGGATAGTTAATGATCAGCCCACTGACGCGTTGCGCGAGAAGATTGTGCACCGCCGCTTTACAGGCTTCGACGCCGCTTCGTTCTACCATCGACACCACCACGCTGGCACCCAGTTGATCGGCGCGAGATTTAATCGCCGCGACAATTTGCGACGGCGCGTGCAGGGCCAGACTGGAGGTGGCAACGCCAATCAGCAACGACTGTTTGCCCGCCAGTTGTTGTGCCACGCGGTTGGGAATGTAATTCAGCTCCGCCATCGCCGCTTCCACTTTTTCCCGCGTTTTCGCAGAAACGTGGCTGGCCTGGTTCACCACGCGGGAAACGGTCTGATAAGAGACACCGGCATACTCTGCGACATCGTATAACGTTACTGGTTTCATATTCACCACCCTGAATTGACTCTCTTCCGGGCGCTATCATGCCATACCGCGAAAGGTTTTGCGCCATTCGATGGCGCGCCGC
Inducible promoter(P_{TAC})	ttcgtcaggccacatagctttcttgttctgatcggaacgatcgttggctgtgTTGACAATTAATCATCGGCTCGTATAATGtgtggAATTGTGAGCGCTCACAATT
K1.5RNAP	attattttattgccactaaggaggtttttcgGTGCAGGGCCTGCACGCCATCCAGCTGCAGCTGGAGGAGGAGATGTTCAACGGCGGCATCCGGAGATTTGA

	GGCAGACCAGCAGAGGCAGATCGCCAGCGGCAACGAGTCCGATA<br>CCGCCTGGAATAGGCGCCTGCTGAGCGAGCTGATCGCCCCAATG<br>GCCGAGGGCATCCAGGCCTACAAGGAGGAGTATGAGGGCAAGC<br>GGGGCAGAGCACCAAGAGCCCTGGCCTTCATCAACTGCGTGGAG<br>AATGAGGTGGCCGCCTACATCACCATGAAGATCGTGATGGACAT<br>GCTGAACACCGATGTGACACTGCAGGCCATCGCCATGAATGTGG<br>CCGATAGGATCGAGGACCAGGTGCGCTTCTCCAAGCTGGAGGGA<br>CACGCAGCCAAGTACTTTGAGAAGGTGAAGAAGTCTCTGAAGGC<br>CAGCAAGACCAAGTCCTATAGGCACGCACACAACGTGGCAGTGG<br>TGGCAGAGAAGTCTGTGGCCGACCGGGATGCCGACTTCAGCAGA<br>TGGGAGGCCTGGCCAAAGGACACCCTGCTGCAGATCGGCATGAC<br>ACTGCTGGAGATCCTGGAGAACAGCGTGTTCTTCAATGGCCAGCC<br>CGTGTTCCTGCGGACCCTGAGAACAAATGGCGGCAAGCACGGCG<br>TGTACTATCTGCAGACCTCCGAGCACGTGGGCGAGTGGATCACA<br>GCCTTTAAGGAGCACGTGGCACAGCTGTCTCCAGCATACGCACCT<br>TGCGTGATCCCACCTAGGCCATGGGTGAGCCCCTTCAACGGAGG<br>ATTTCACACCGAGAAGGTGGCCTCCAGGATCCGCCTGGTGAAGG<br>GCAATAGGGAGCACGTGCGCAAGCTGACCAAGAAGCAGATGCCC<br>GAGGTGTACAAGGCCGTGAACGCCCTGCAGGCCACAAAGTGGCA<br>AGTGAATAAGGAGGTGCTGCAGGTGGTGGAGGACGTGATCAGGC<br>TGGATCTGGGCTATGGCGTGCCTTCCTTCAAGCCACTGATCGACC<br>GCGAGAACAAGCCTGCCAATCCAGTGCCCCTGGAGTTTCAGCAC<br>CTGCGGGGCAGAGAGCTGAAGGAGATGCTGACCCCAGAGCAGTG<br>GCAGGCCTTCATCAACTGGAAGGGCGAGTGTACAAAGCTGTACA<br>CCGCCGAGACAAAGAGGGGCTCTAAGAGCGCCGCAACCGTGAGG<br>ATGGTGGGACAGGCCCGCAAGTACTCTCAGTTCGACGCCATCTAC<br>TTCGTGTACGCCCTGGATTCCAGGTCTCGCGTGTATGCCCAGAGC<br>TCCACCCTGTCTCCTCAGAGCAATGACCTGGGCAAGGCCCTGCTG<br>CGGTTCACAGAGGGCCAGAGACTGGATAGCGCCGAGGCCCTGAA<br>GTGGTTTCTGGTGAACGGCGCCAACAATTGGGGCTGGGACAAGA<br>AGACCTTCGATGTGCGGACAGCCAATGTGCTGGACAGCGAGTTT<br>CAGGATATGTGCAGAGACATCGCCGCCGATCCCCTGACCTTCACA<br>CAGTGGGTGAACGCCGATTCCCCTTATGGCTTCCTGGCCTGGTGT<br>TTTGAGTACGCCAGGTATCTGGATGCCCTGGACGAGGGCACCCA<br>GGACCAGTTTATGACACACCTGCCAGTGCACCAGGATGGCAGCT<br>GCTCCGGCATCCAGCACTACAGCGCCATGCTGAGGGACGCAGTG<br>GGAGCCAAGGCCGTGAATCTGAAGCCCTCTGACAGCCCTCAGGA<br>TATCTATGGCGCCGTGGCCCAGGTGGTCATCCAGAAGAACTACG

	CCTATATGAATGCCGAGGACGCCGAGACCTTCACATCCGGATCTG TGACCCTGACAGGAGCAGAGCTGCGGTCCATGGCCTCTGCCTGG GATATGATCGGCATCACCAGAGGCCTGACAAAGAAGCCAGTGAT GACCCTGCCATACGGAAGCACCAGGCTGACATGTAGAGAGTCCG TGATCGACTATATCGTGGATCTGGAGGAGAAGGAGGCACAGAGG GCAATCGCAGAGGGAAGGACAGCAAACCCTGTGCACCCATTTGA TAATGACAGGAAGGACTCTCTGACCCCAAGCGCCGCCTACAACT ATATGACAGCCCTGATCTGGCCTAGCATCTCCGAGGTGGTGAAG GCCCCAATCGTGGCCATGAAGATGATCAGGCAGCTGGCCAGGTT CGCAGCAAAGAGGAATGAGGGCCTGGAGTACACCCTGCCAACAG GCTTTATCCTGCAGCAGAAGATCATGGCCACCGATATGCTGAGA GTGTCCACATGCCTGATGGGCGAGATCAAGATGTCTCTGCAGATC GAGACCGATGTGGTGGACGAGACAGCAATGATGGGAGCTGCCGC CCCAAACTTCGTGCACGGACACGACGCCTCCCACCTGATCCTGAC CGTGTGCGATCTGGTGGACAAGGGCATCACATCCATCGCCGTGAT CCACGATTCTTTTGGCACCCACGCAGGAAGAACAGCAGATCTGA GGGACTCTCTGAGAGCCGAGATGGTGAAGATGTACCAGGGCAGG AACGCCCTGCAGAGCCTGCTGGACGAGCACGAGGAGCGGTGGCT GGTGGATACCGGCATCCAGGTGCCTGAGCAGGGCGAGTTCGACC TGAATGAGATCCTGGTGAGCGATTATTGTTTTGCCTAA
K1.5 promoter	tggtcctactagatgcctccacaccgctcgtcacatcctgggcctccacggccTAATCAGTATTT ACTGGACACTATAGAAGGG

Table S2 Sequences of Crucial Parts of the K1.5 promoter-reporter and K1.5 RNAP Backbones in Mammalian Cells

core insulator	agggacagcccccccccaaagcccccagggatgtaattacgtccctcccccgctagggggcagcagcga gccgcccggggctccgctccggtccggcgctcccccgcatccccgagccggcagcgtgcggggacag cccgggcacggggaaggtggcacgggatcgctttcctctgaacgcttctc
K1.5 core promoter	TAATCAGTATTTACTGGACACTATAGAAGGG
TRE3G promoter	TAGGCGTGTACGGTGGGCGCCTATAAAAGCAGAGCTCGTTTAGTG AACCGTCAGATCGCCTGGAGCAATTCCACAACACTTTTGTCTTAT ACCAACTTTCCGTACCACTTCCTACCCTCGTAAA
Citrine (reporter gene)	ATGCCACCATGGTGAGCAAGGGCGAGGAGCTGTTCACCGGGGTG GTGCCCATCCTGGTCGAGCTGGACGGCGACGTAAACGGCCACAA GTTCAGCGTGTCCGGCGAGGGCGAGGGCGATGCCACCTACGGCA AGCTGACCCTGAAGTTCATCTGCACCACCGGCAAGCTGCCCGTGC CCTGGCCCACCCTCGTGACCACCTTCGGCTACGGCCTGATGTGCTT

	CGCCCGCTACCCCGACCACATGAAGCAGCACGACTTCTTCAAGTC CGCCATGCCCGAAGGCTACGTCCAGGAGCGCACCATCTTCTTCAA GGACGACGGCAACTACAAGACCCGCGCCGAGGTGAAGTTCGAGG GCGACACCCTGGTGAACCGCATCGAGCTGAAGGGCATCGACTTCA AGGAGGACGGCAACATCCTGGGGCACAAGCTGGAGTACAACTAC AACAGCCACAACGTCTATATCATGGCCGACAAGCAGAAGAACGG CATCAAGGTGAACTTCAAGATCCGCCACAACATCGAGGACGGCA GCGTGCAGCTCGCCGACCACTACCAGCAGAACACCCCCATCGGCG ACGGCCCCGTGCTGCTGCCCGACAACCACTACCTGAGCTACCAGT CCGCCCTGAGCAAAGACCCCAACGAGAAGCGCGATCACATGGTC CTGCTGGAGTTCGTGACCGCCGCCGGGATCACTCTCGGCATGGAC GAGCTGTACAAGTAA
EF1a promoter	ggaatcccagggaccgtcgttaaactcccactaacgtagaacccagagatcgctgcgttcccgccccctcac ccgcccgctctcgtcatcactgaggtggataagagcatgcgtgaggctccggtgcccgtcagtgggcagag cgcacatcgcccacagtccccgagaagttggggggaggggtcggcaattgaaccggtgcctagagaaggt ggcgcggggtaaactgggaaagtgatgtcgtgtactggctccgcctttttcccgagggtgggggagaaccgt atataagtgcagtagtcgccgtgaacgttctttttcgcaacgggtttgccgccagaacacaggtaagtgccgtg tgtggttcccgcgggcctggcctctttacgggttatggcccttgcgtgccttgaattacttccacgcccctggct gcagtacgtgattcttgatcccgagcttcgggttggaagtgggtgggagagttcgaggccttgcgcttaagga gccccttcgcctcgtgcttgagttgaggcctggcttgggcgctggggccgccgcgtgcgaatctggtggcac cttcgcgcctgtctcgctgctttcgataagtctctagccatttaaaatttttgatgacctgctgcgacgctttttttct ggcaagatagtcttgtaaatgcgggccaagatctgcacactggtatttcggtttttggggccgcgggcggcga cggggcccgtgcgtcccagcgcacatgttcggcgaggcggggcctgcgagcgcggccaccgagaatcg gacgggggtagtctcaagctggccggcctgctctggtgcctggcctcgcgccgccgtgtatcgccccgccc tgggcggcaaggctggcccggtcggcaccagttgcgtgagcggaaagatggccgcttcccggccctgctg cagggagctcaaaatggaggacgcggcgctcgggagagcgggcgggtgagtcacccacacaaaggaaa agggcctttccgtcctcagccgtcgcttcatgtgactccacggagtaccgggcgccgtccaggcacctcgatt agttctcgagcttttggagtacgtcgtctttaggttggggggaggggttttatgcgatggagtttccccacactg agtgggtggagactgaagttaggccagcttggcacttgatgtaattctccttggaatttgccctttttgagtttgg atcttggttcattctcaagcctcagacagtggttcaaagtttttttcttccatttcaggtgtcgtga
Capping enzyme-K1.5 RNAP	ATGGCCTCCCTGGACAATCTGGTGGCCCGGTACCAGAGATGCTTT AATGATCAGTCTCTGAAGAACAGCACCATCGAGCTGGAGATCAG ATTCCAGCAGATCAACTTCCTGCTGTTTAAGACCGTGTATGAGGC CCTGGTGGCCCAGGAGATCCCTTCTACAATCTCTCACAGCATCAG ATGCATCAAGAAGGTGCACCACGAGAATCACTGTAGGGAGAAGA TCCTGCCAAGCGAGAACCTGTACTTTAAGAAGCAGCCTCTGATGT TCTTTAAGTTCTCCGAGCCAGCCTCTCTGGGCTGTAAGGTGAGCCT GGCCATCGAGCAGCCTATCAGGAAGTTTATCCTGGACAGCTCCGT

	GCTGGTGCGGCTGAAGAACAGAACCACATTCAGGGTGTCCGAGC<br>TGTGGAAGATCGAGCTGACCATCGTGAAGCAGCTGATGGGCTCTG<br>AGGTGAGCGCCAAGCTGGCAGCCTTCAAGACCCTGCTGTTTGACA<br>CACCCGAGCAGCAGACCACAAAGAATATGATGACACTGATCAAC<br>CCTGACGATGAGTACCTGTATGAGATCGAGATCGAGTACACCGGC<br>AAGCCAGAGTCCCTGACAGCAGCAGATGTGATCAAGATCAAGAA<br>TACCGTGCTGACACTGATCTCTCCCAACCACCTGATGCTGACCGC<br>CTATCACCAGGCCATCGAGTTTATCGCCTCTCACATCCTGTCTAGC<br>GAGATCCTGCTGGCCAGAATCAAGAGCGGCAAGTGGGGCCTGAA<br>GAGGCTGCTGCCACAGGTGAAGTCCATGACCAAGGCCGATTACAT<br>GAAGTTCTATCCCCCTGTGGGCTACTATGTGACCGACAAGGCCGA<br>TGGCATCCGCGGCATCGCCGTGATCCAGGACACACAGATCTACGT<br>GGTGGCCGATCAGCTGTATAGCCTGGGCACCACAGGCATCGAGCC<br>ACTGAAGCCCACCATCCTGGACGGCGAGTTTATGCCCGAGAAGA<br>AGGAGTTCTACGGCTTTGATGTGATCATGTATGAGGGCAATCTGC<br>TGACCCAGCAGGGCTTCGAGACACGGATCGAGTCCCTGTCTAAGG<br>GCATCAAGGTGCTGCAGGCCTTTAACATCAAGGCCGAGATGAAG<br>CCCTTCATCTCCCTGACCTCTGCCGACCCTAACGTGCTGCTGAAGA<br>ATTTCGAGAGCATCTTCAAGAAGAAGACCCGCCCTTACTCCATCG<br>ATGGCATCATCCTGGTGGAGCCAGGCAATTCCTATCTGAACACCA<br>ATACCTTCAAGTGGAAGCCAACCTGGGACAATACACTGGATTTCC<br>TGGTGCGGAAGTGCCCCGAGTCTCTGAACGTGCCTGAGTACGCCC<br>CAAAGAAGGGCTTTTCTCTGCACCTGCTGTTCGTGGGCATCAGCG<br>GCGAGCTGTTTAAGAAGCTGGCCCTGAACTGGTGTCCAGGCTACA<br>CCAAGCTGTTCCCCGTGACACAGAGAAACCAGAATTATTTCCCCG<br>TGCAGTTTCAGCCCTCTGACTTCCCTCTGGCCTTTCTGTACTATCA<br>CCCTGACACCTCCTCTTTCAGCAATATCGATGGCAAGGTGCTGGA<br>GATGCGCTGCCTGAAGCGGGAGATCAACTATGTGAGGTGGGAGA<br>TCGTGAAGATCAGGGAGGACCGCCAGCAGGATCTGAAGACAGGC<br>GGCTACTTCGGCAATGACTTTAAGACCGCCGAGCTGACATGGCTG<br>AACTATATGGACCCCTTCAGCTTCGAGGAGCTGGCCAAGGGCCCC<br>TCTGGCATGTACTTTGCCGGCGCCAAGACCGGCATCTATAGGGCC<br>CAGACAGCCCTGATCTCTTTCATCAAGCAGGAGATCATCCAGAAG<br>ATCAGCCACCAGTCCTGGGTCATCGACCTGGGAATCGGCAAGGG<br>ACAGGACCTGGGCAGATACCTGGATGCAGGCGTGAGGCACCTGG<br>TGGGAATCGACAAGGATCAGACCGCCCTGGCAGAGCTGGTGTAC<br>AGGAAGTTCAGCCACGCCACCACACGGCAGCACAAGCACGCCAC<br>AAATATCTATGTGCTGCACCAGGATCTGGCCGAGCCTGCCAAGGA

	GATCAGCGAGAAGGTGCACCAGATCTACGGCTTTCCAAAGGAGG<br>GCGCCAGCTCCATCGTGTCCAACCTGTTCATCCACTATCTGATGA<br>AGAATACCCAGCAGGTGGAGAACCTGGCCGTGCTGTGCCACAAG<br>CTGCTGCAGCCAGGAGGAATGGTGTGGTTCACCACAATGCTGGGC<br>GAGCAGGTGCTGGAGCTGCTGCACGAGAACCGCATCGAGCTGAA<br>TGAAGTGTGGGAGGCCCGGGAGAACGAGGTGGTGAAGTTTGCCA<br>TCAAGCGCCTGTTCAAGGAGGACATCCTGCAGGAGACCGGACAG<br>GAGATCGGCGTGCTGCTGCCCTTCTCCAACGGCGACTTCTACAAT<br>GAGTATCTGGTGAACACAGCCTTCCTGATCAAGATCTTTAAGCAC<br>CACGGCTTCAGCCTGGTGCAGAAGCAGTCCTTCAAGGACTGGATT<br>CCCGAGTTCCAGAACTTCAGCAAGTCCCTGTACAAGATCCTGACC<br>GAGGCCGATAAGACCTGGACAAGCCTGTTCGGCTTTATCTGTCTG<br>CGGAAGAACggaggaggaggtagtggaggaggaggtagtggcggaggcggtagcggaggcgg<br>cggtagcCAGGGCCTGCACGCCATCCAGCTGCAGCTGGAGGAGGAGA<br>TGTTCAACGGCGGCATCCGGAGATTTGAGGCAGACCAGCAGAGG<br>CAGATCGCCAGCGGCAACGAGTCCGATACCGCCTGGAATAGGCG<br>CCTGCTGAGCGAGCTGATCGCCCCAATGGCCGAGGGCATCCAGGC<br>CTACAAGGAGGAGTATGAGGGCAAGCGGGGCAGAGCACCAAGA<br>GCCCTGGCCTTCATCAACTGCGTGGAGAATGAGGTGGCCGCCTAC<br>ATCACCATGAAGATCGTGATGGACATGCTGAACACCGATGTGACA<br>CTGCAGGCCATCGCCATGAATGTGGCCGATAGGATCGAGGACCA<br>GGTGCGCTTCTCCAAGCTGGAGGGACACGCAGCCAAGTACTTTGA<br>GAAGGTGAAGAAGTCTCTGAAGGCCAGCAAGACCAAGTCCTATA<br>GGCACGCACACAACGTGGCAGTGGTGGCAGAGAAGTCTGTGGCC<br>GACCGGGATGCCGACTTCAGCAGATGGGAGGCCTGGCCAAAGGA<br>CACCCTGCTGCAGATCGGCATGACACTGCTGGAGATCCTGGAGAA<br>CAGCGTGTTCTTCAATGGCCAGCCCGTGTTCCTGCGGACCCTGAG<br>AACAAATGGCGGCAAGCACGGCGTGTACTATCTGCAGACCTCCG<br>AGCACGTGGGCGAGTGGATCACAGCCTTTAAGGAGCACGTGGCA<br>CAGCTGTCTCCAGCATACGCACCTTGCGTGATCCCACCTAGGCCA<br>TGGGTGAGCCCCTTCAACGGAGGATTTCACACCGAGAAGGTGGCC<br>TCCAGGATCCGCCTGGTGAAGGGCAATAGGGAGCACGTGCGCAA<br>GCTGACCAAGAAGCAGATGCCCGAGGTGTACAAGGCCGTGAACG<br>CCCTGCAGGCCACAAAGTGGCAAGTGAATAAGGAGGTGCTGCAG<br>GTGGTGGAGGACGTGATCAGGCTGGATCTGGGCTATGGCGTGCCT<br>TCCTTCAAGCCACTGATCGACCGCGAGAACAAGCCTGCCAATCCA<br>GTGCCCCTGGAGTTTCAGCACCTGCGGGGCAGAGAGCTGAAGGA<br>GATGCTGACCCCAGAGCAGTGGCAGGCCTTCATCAACTGGAAGG

	GCGAGTGTACAAAGCTGTACACCGCCGAGACAAAGAGGGGCTCT<br>AAGAGCGCCGCAACCGTGAGGATGGTGGGACAGGCCCGCAAGTA<br>CTCTCAGTTCGACGCCATCTACTTCGTGTACGCCCTGGATTCCAGG<br>TCTCGCGTGTATGCCCAGAGCTCCACCCTGTCTCCTCAGAGCAAT<br>GACCTGGGCAAGGCCCTGCTGCGGTTCACAGAGGGCCAGAGACT<br>GGATAGCGCCGAGGCCCTGAAGTGGTTTCTGGTGAACGGCGCCA<br>ACAATTGGGGCTGGGACAAGAAGACCTTCGATGTGCGGACAGCC<br>AATGTGCTGGACAGCGAGTTTCAGGATATGTGCAGAGACATCGCC<br>GCCGATCCCCTGACCTTCACACAGTGGGTGAACGCCGATTCCCCT<br>TATGGCTTCCTGGCCTGGTGTTTTGAGTACGCCAGGTATCTGGATG<br>CCCTGGACGAGGGCACCCAGGACCAGTTTATGACACACCTGCCAG<br>TGCACCAGGATGGCAGCTGCTCCGGCATCCAGCACTACAGCGCCA<br>TGCTGAGGGACGCAGTGGGAGCCAAGGCCGTGAATCTGAAGCCC<br>TCTGACAGCCCTCAGGATATCTATGGCGCCGTGGCCCAGGTGGTC<br>ATCCAGAAGAACTACGCCTATATGAATGCCGAGGACGCCGAGAC<br>CTTCACATCCGGATCTGTGACCCTGACAGGAGCAGAGCTGCGGTC<br>CATGGCCTCTGCCTGGGATATGATCGGCATCACCAGAGGCCTGAC<br>AAAGAAGCCAGTGATGACCCTGCCATACGGAAGCACCAGGCTGA<br>CATGTAGAGAGTCCGTGATCGACTATATCGTGGATCTGGAGGAGA<br>AGGAGGCACAGAGGGCAATCGCAGAGGGAAGGACAGCAAACCC<br>TGTGCACCCATTTGATAATGACAGGAAGGACTCTCTGACCCCAAG<br>CGCCGCCTACAACTATATGACAGCCCTGATCTGGCCTAGCATCTC<br>CGAGGTGGTGAAGGCCCCAATCGTGGCCATGAAGATGATCAGGC<br>AGCTGGCCAGGTTCGCAGCAAAGAGGAATGAGGGCCTGGAGTAC<br>ACCCTGCCAACAGGCTTTATCCTGCAGCAGAAGATCATGGCCACC<br>GATATGCTGAGAGTGTCCACATGCCTGATGGGCGAGATCAAGATG<br>TCTCTGCAGATCGAGACCGATGTGGTGGACGAGACAGCAATGAT<br>GGGAGCTGCCGCCCCAAACTTCGTGCACGGACACGACGCCTCCCA<br>CCTGATCCTGACCGTGTGCGATCTGGTGGACAAGGGCATCACATC<br>CATCGCCGTGATCCACGATTCTTTTGGCACCCACGCAGGAAGAAC<br>AGCAGATCTGAGGGACTCTCTGAGAGCCGAGATGGTGAAGATGT<br>ACCAGGGCAGGAACGCCCTGCAGAGCCTGCTGGACGAGCACGAG<br>GAGCGGTGGCTGGTGGATACCGGCATCCAGGTGCCTGAGCAGGG<br>CGAGTTCGACCTGAATGAGATCCTGGTGAGCGATTATTGTTTTGC<br>CTAA
βGH poly signal	gcctcgactgtgccttctagttgccagccatctgttgtttgcccctcccccgtgccttccttgaccctggaaggtg<br>ccactcccactgtcctttcctaataaaatgaggaaattgcatcgcattgtctgagtaggtgtcattctattctgggg<br>ggtggggtggggcaggacagcaagggggaggattgggaagacaatagcaggcatgctgggga

Table S3 Specific promoter optimization route used in fig 1b

Route number	Sequence (start from the first sequence)
route_1_seq	ACCAAAAGGTTACTAGGACAGTACCAACGGT<br>ACCAAAAGGTTTCTAGGACAGTACCGACGGT<br>ACCAAAAGGTTTCTAGGACAGTACAGACGGT<br>ACCAAAAGGTTTCTAGGACAGTATAGACGGT<br>ACCAAAAGGTTTCTAGGACAGTATAGAAGGT<br>ACCAAAAGGTTTATAGGACAGTATAGAAGGT<br>ACCAAAATGTTTATAGGACAGTATAGAAGGT
route_2_seq	AGGATATGGTATCTAGGACAGTAGTGACGAG<br>AGGATATGGTTTCTAGGACAGTATTGACGAG<br>AGGATATGGTTTATAGGACAGTATTGAAGAG<br>AGGATATGGTTTATAGGACAGTATAGAAGAG<br>AGGATATTGTTTATAGGACAGTATAGAAGAG<br>AGGATATTGTTTATAGGACACTATAGAAGAG<br>AGGATGTTATTTATAGGACACTATAGAAGAG<br>AGGATGTTATTTACAGGACACTATAGAAGAG
route_3_seq	TCGGTTACTGTTCCTTGACAGTATGGGATAC<br>TCGGTTACTGTTCCTTGACAGTATAGGATAC<br>TCGGGTACTGTTACTGGACAGTATAGAACAC<br>TCGGGGACTGTTACTGGACAGTATAGAACAC<br>TCGGGGACTTTTACTGGACAGTATAGAACAC
route_4_seq	GGTTCTATCCTTAAGCGACAGTCAAGGGCGC<br>GGTTCTATCCTTAAGGGACAGTCAAGGGCGC<br>GGTTCTATCCTTACTGGACAGTCTAGGGCGC<br>GGTTCTATCTTTACTGGACAGTCTAGGGCGC<br>GGTTCTATCTTTACTGGACAGTCTAGAGCGC<br>GGTTCTATCTTTACTGGACAGTCTAGAACGC

Table S4 Semi-rational designed sequences used in Fig 4e,f

number	sequence
1	CAAACGCAATATAACTGACACTATAGAATAT
2	GTATCGGCCTTTAACGGACACTATAGAAATA
3	CACAGCGCGTTTACCGGACACTATAGGACAG
4	TTTTTAATATTTAGCGGACACTCTAGAATGT
5	GTATAGGAACTTACAGGACACTATAGAACGC
6	CTGGCCTCAATTAGTGGACACTATAGATGTG

7	TTAGTGGTATCTAGCGGACACTATAGAAGAA
8	GAACCCCTCTTTAGTGGACACTCTAGAACGG
9	TTCTGCGTACTTACTAGACACTATAGAAGAT
10	TAGCTACCATTTAATTGACACTATAGAAGTG
11	GGAGGACTATTTAGAGGACACTATAGACCGC
12	TGTTTTGTAATTAGTGGACACTCTAGAATGC

Table S5 primer used for random K1.5 promoter library and primers for promoters' library identification.

name	sequence
K1.5-21N-F	CTGggcctccacggccTAATNNNNNNNNNNNNNNNNNNNNNGAAGGGAGCTGTCACCGGATGTGC
K1.5-21N-R	ATTAggccgtggaggccCAGGATGTGACGAGC
PK1.5-F	atagctcttcaTGGTCCtactagATGCCTC
PK1.5-R	AtagctcttcaGCT
PK1.5-P-F	agtactgtgatgagtggcagg
PK1.5-P-R	cttaaaggaaatcgtgcgttcc

Table S6 The optimization route used in Fig. 5f.

**route_1_seq**	**route_2_seq**
GCCGTGTGAGGTAGACGACGTGGGATTGTGC<br>GCCGTGTGAGTTAGACGACGTGGGATTGTGC<br>GCCGTGTGAGTTAGAGGACATGGGAGTGTGC<br>GCCGTGTGAGTTAGAGGACATTCGAGTGTGC<br>GCCGTGTGAGTTAGAGGACATTCGAGAGTGC<br>GCCGTGTGAGTTAGAGGACATTCTAGAGTGC<br>GCCGTGTGAGTTAGAGGACACTCTAGAGTGC<br>GCCGTGTGAGTTAGAGGACACTCTAGAATGC<br>GCCGTGTTAGTTAGAGGACACTCTAGAATGC<br>GCCGTGTTATTTAGAGGACACTCTAGAATGC<br>GCCGTGTTATTTAGAGGACACTCTAGAAGGC<br>GCCGTGTTATTTAGTGGACACTCTAGAAGGC	GGTTCTATCCCTAAGCGATGGTCAACGGCGC<br>GGTTCTATCCCTAAGCGACGGTCAACGGCGC<br>GGTTCTATCCTTAAGCGACAGTCAACGGCGC<br>GGTTCTATCCTTAAGCGACAGTCAAGGGCGC<br>GGTTCTATCCTTAAGGGACAGTCAAGGGCGC<br>GGTTCTATCCTTACTGGACAGTCTAGGGCGC<br>GGTTCTATCTTTACTGGACAGTCTAGGGCGC<br>GGTTCTATCTTTACTGGACAGTCTAGAGCGC<br>GGTTCTATCTTTACTGGACAGTCTAGAACGC
**route_3_seq**	**route_4_seq**
GTGCACAGGGATGGTCAACATCACTGGGCGG<br>GTGCACAGGGATGGTCAACATTACTGGGCGG<br>GTGCACAGGGATGGTCGACATTACTGGGCGG<br>GTGCACAGGGATGGTCGACATTACTGGACGG<br>GTGCACAGGGATGGTCGACATTATTGGACGG<br>GTGCACAGGGATGGTCGACATTATAGGACGG	CTAGGGCAATGCAGCAACTAGGATAATCACG<br>CTAGGGCAATGCAGCAACTAGTATAATCACG<br>CTAGGGCAATGCAGCAACCAGTATAATCACG<br>CTAGGGCAATTCAGCAACCAGTATAATCACG<br>CTAGGGCAATTCAGCAGCCAGTATAATCACG<br>CTAGGGCAATTCAGCAGCCAGTATAGTCACG

GTGCACAGGGATAGTCGACATTATAGGACGG<br>GTGCACAGGGATAGTCGACACTATAGGACGG<br>GTGCACAGGGATAGTGGACACTATAGGACGG<br>GTGCACAGGGTTAGTGGACACTATAGGACGG<br>GTGCACAGGGTTAGTGGACACTATAGAACGG<br>GTGCAGAGGGTTAGTGGACACTATAGAACGG<br>GTGCGGAGGGTTAGTGGACACTATAGAACGG<br>GTGCGGACGGTTAGTGGACACTATAGAACGG	CTAGGGCAATTTAGCAGCCAGTATAGTCACG<br>CTAGGGCAATTTAGCAGCCAGTATAGACACG<br>CTAGGGCAATTTAGCAGCCAGTATAGAAACG<br>CTAGGGCAATTTAGCAGCCAGTATAGAAGCG<br>CTAGGGCAATTTAGCAGACAGTATAGAAGCG
**route_5_seq**<br>ACGGTGTTAGAGCAGTGTCCCTCACAGCTCC<br>ACGGTGTTAGAGCAGTGTCACTCACAGCTCC<br>ACGGTGTTAGAACAGTGCCACTCACAGCTCC<br>ACGGTGTTAGAACAGTGCCACTCTCAGCTCC<br>ACGGTGTTAGAACAGTGCCACTCTAGGCTCC<br>ACGGTGTTAGAACAGTGCCACTCTAGAATCC<br>ACGGTGTTAGAACGGTGCCACTCTAGAATCC<br>ACGGTGTTTGAACGGTGCCACTCTAGAATCC<br>ACGGTGTTTGAACGGTGCCACTCTAGAACCC	**route_6_seq**<br>GGACGTGTAATTATAGAACGTGATTAGCATC<br>GGACGTGTAATTATAGAACGTTATTAGCATC<br>GGACGTGTAATTATAGAACGTTATTGGCATC<br>GGACGTGTATTTATAGAACGTTATTGGCATC<br>GGACGTGTATTTATAGAACGTTATAGGCATC<br>GGACGTGTATTTATAGAACATTATAGGCATC<br>GGACGTGTATTTATAGAACATTATAGACATC<br>GGACGTGTATTTATAGAACATTATAGAAATC<br>GGACGTGTATTTATAGGACATTATAGAAATC<br>GGACGTGTATTTATAGGACATTATAGAACTC<br>GGACGTGTATTTAGAGGACATTATAGAACTC<br>GGACGGGTATTTAGAGGACATTATAGAACTC
**route_7_seq**<br>GAGCGCGCGAAAAGGCGCCTACGTCCTATAC<br>GAGCGCGCGAAAAGGCGCCTACCTCCTATAC<br>GAGCGCGCGAAAAGGCGCCTACCTCATATAC<br>GAGCGCGCGAAAAGGCGCCTATCTCGTATAC<br>GAGCGCGCGAAAAGGCGCCAATCTCGTATAC<br>GAGCGCGCGAAAAGGCGCCAGTCTCGTATAC<br>GAGCGCGCGAAAAGGCGCCAGTCTAGTATAC<br>GAGCGGGCGAAAAGGAGCCAGTCTAGTACAC<br>GAGCGGGCGATAAGGAGCCAGTCTAGAACAC<br>GAGCGGGCGATAAGGGGCCAGTCTAGAACAC<br>GAGCGGGCGATAAGGGGCCACTCTAGAACAC	**route_8_seq**<br>GAGACAACAAAGTCGGTCATTACACGGTTGT<br>GAGACAACAAAGTCGGTCATTACAGGGTTGT<br>GAGACAACAAAGTCGGACATTACAGGGTTGT<br>GAGACAACAAAATCGGACATTACAGGGTTGT<br>GAGACAACAAAATCGGACATTATAGGGTTGT<br>GAGACAACAAAATCGGACAGTATAGGGTTGT<br>GAGACAACAAAATCGGACAGTATAGGATTGT<br>GAGACGACAAAATCGGACAGTATAGGATTGT<br>GAGACGACAAAATCGTACAGTATAGGATTGT<br>GAGACGACAATATCGTACAGTATAGGATTGT<br>GAGACGACTATATCGTACAGTATAGGATTGT<br>GAGACGACTATATGGTACAGTATAGAATTGT
**route_9_seq**<br>CCGTGCGGGGGTGCTTTTACTATTCGGAAAG<br>CCGTGCGGGGGTGCTTTTAGTATTCGGAAAG<br>CCGTGCGGGGGTGCTTTTAGTATTGGGAAAG<br>CCGTGCGGGGGTGCTTTTAGTATTGGGACAG<br>CCGTGCGGGGATGCTTTTAGTATTGGGACAG<br>CCGTGCGGGGATGCTTATAGTATTGGGACAG<br>CCGTGCGGGGATGCTTATAGTATAGGGACAG<br>CCGTGCGGGGATGCTTATAGTATAGGAACAG<br>CCGTGCGGGGTTGCTTATAGTATAGGAACAG	**route_10_seq**<br>AGCACGTCTCCTAACCATTGTTGTATTCTAA<br>AGCACGTCTCCTAACCATTGTTCTATTCTAA<br>AGCACGTCTCCTAACAATTGTTCTAGTCTAA<br>AGCACGTCTCTTAACAATTGTTCTAGTCTAA<br>AGCACGTCTCTTAAGAATTGTTCTAGTCTAA<br>AGCACGTCTCTTAAGAATTGTACTAGTCTAA<br>AGCACGTCTATTTAGAATTGTACTAGTCTAA<br>AGCACGTCTATTTAGAATTGTACTAGACTAA<br>AGCACGTCTATTTAGTATTGTACTAGACTAA

CCGTGCGGGGTTGCGTATAGTATAGGAACAG<br>CCGTGCGGGGTTGGGTATAGTATAGGAACAG<br>CCGTGCGGGGTTTGGTATAGTATAGGAACAG	AGCACGTCTATTTAGTATTGTACTGGACTAA

Table S7. Pearson R-squared correlation between predictions and experimental results (based on three replicate experiments)

Pearson R-squared	Before Purification	After Purification
CNN-1	0.634	0.807
CNN-2	0.488	0.590
CNN-3	0.733	0.887
CNN-LSTM-1	0.807	0.550
CNN-LSTM-2	0.258	0.879
CNN-LSTM-3	0.731	0.666
Attention-1	0.465	0.529
Attention-2	0.625	0.576
Attention-3	0.264	0.705

Supplementary Figures

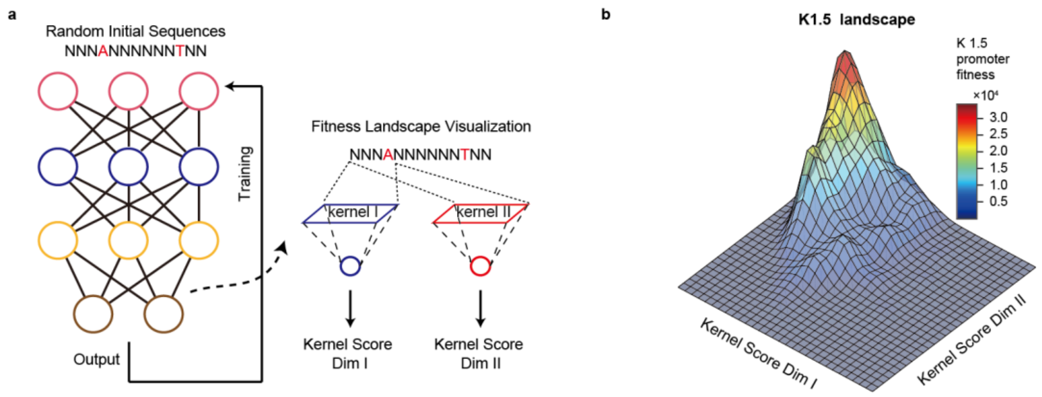

Supplementary Figure 1. Neural network interpretation methods to obtain the gene activity landscape. **a**, the schematic of the landscape construction method. The feature map obtained from the last layer was used to construct the landscape. The first two CNN kernels in the feature map were used to set the two dimensions of the landscape, referred to as Kernel Score Dim I and Kernel Score Dim II. **b**, the landscape of the *De novo* design strategy.

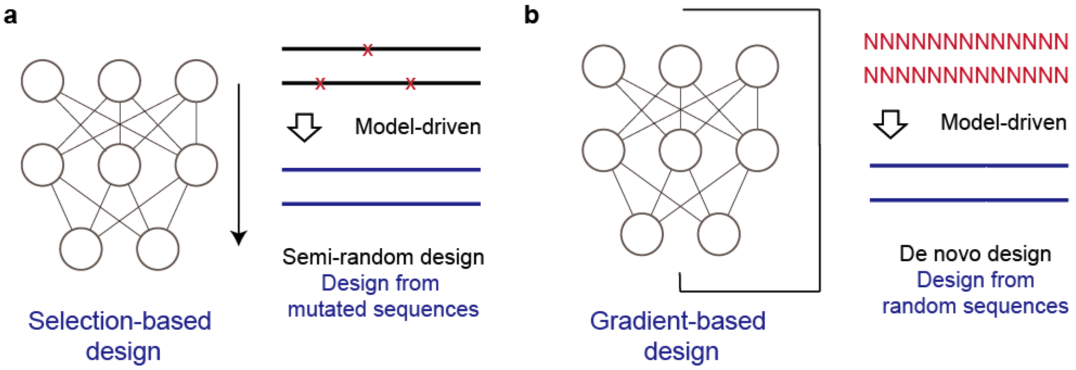

Supplementary Figure 2. Two design strategies are based on filtering methods (Selection-based design) and gradient-based methods. **a**, semi-rational design is based on the filtering method. Potential sequences are mutated from the wild-type functional sequence, and a neural network is used to select the sequences with a predefined gene activity. **b**, *de novo* design is based on gradient-based design. Starting from random sequences, the gradient-based design utilizes the negative gradient direction to gradually adjust the sequences towards the predefined gene activity.

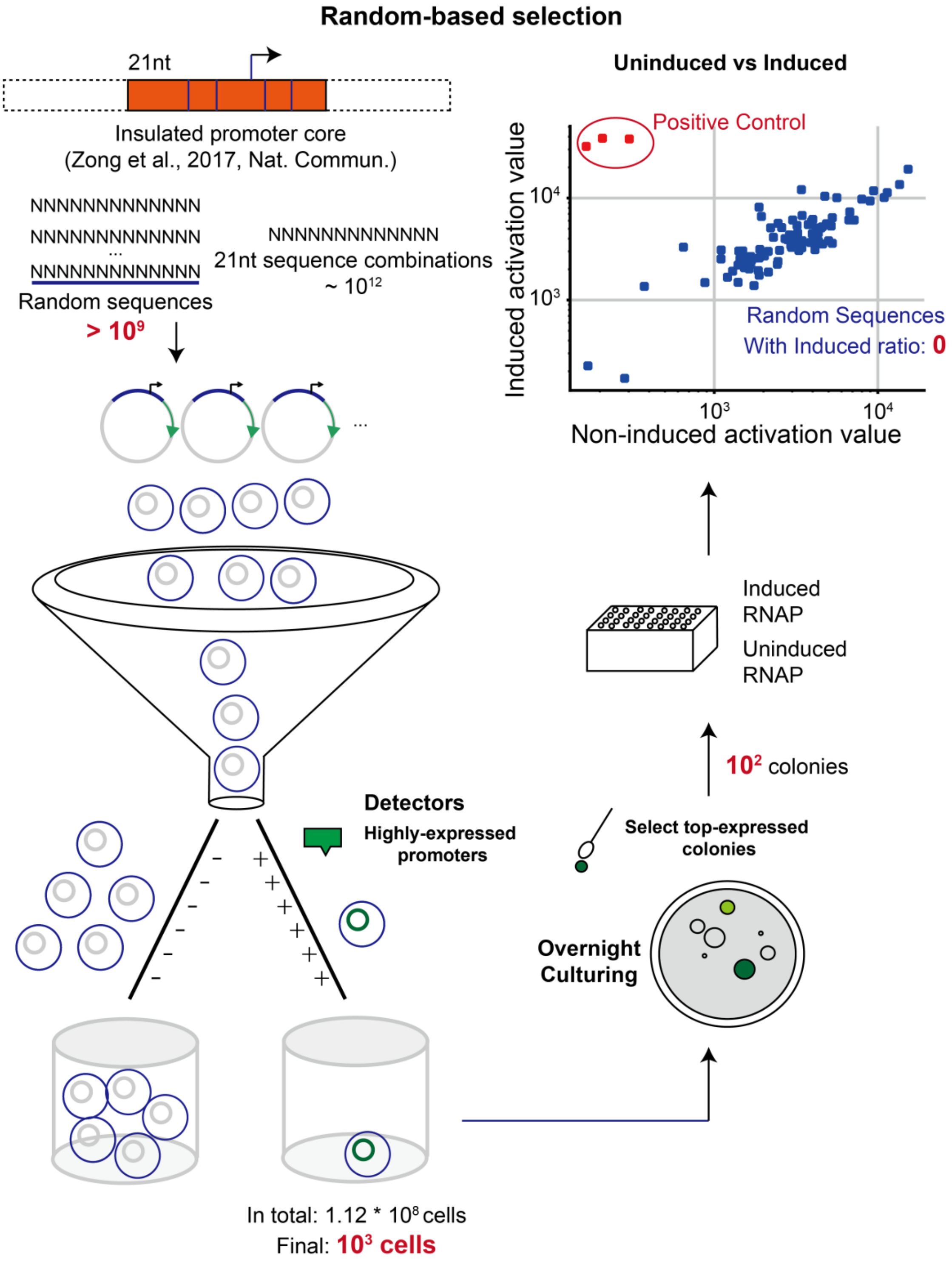

Supplementary Figure 3. Random-based design strategy. The core sequences in the wild-type insulated promoter were substituted with purely random sequences. More than 10^9 random sequences were screened using flow cytometry, followed by serial cultivation, to select random sequences with high expression after induction.

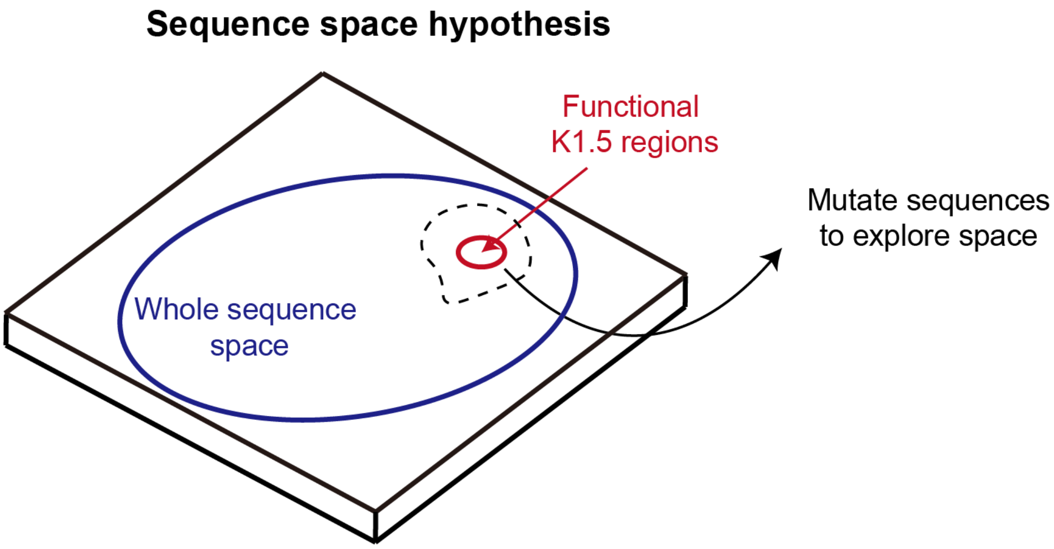

Supplementary Figure 4. Sequence Space Hypothesis. In the entire sequence space, the functional K1.5 regions occupy only a very small portion. Through random mutation, it is possible to identify the boundaries of these regions and discover K1.5 cis-regulatory sequences with specific functions.

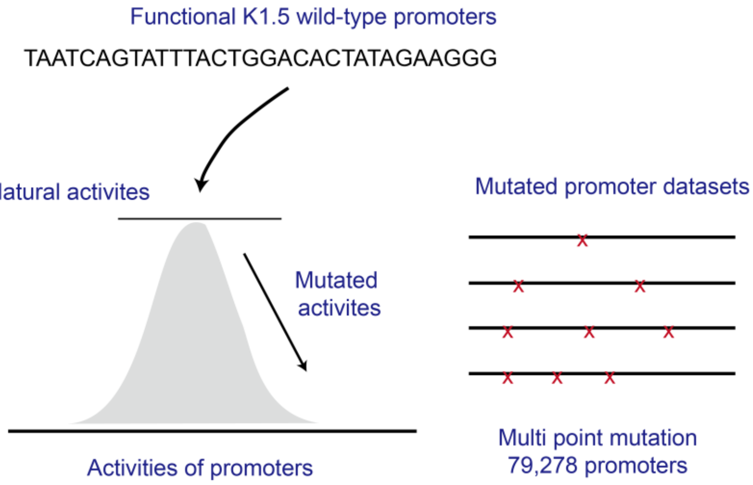

Supplementary Figure 5. Methods to Mutate the Natural K1.5 Sequences. Mutating the K1.5 wild-type promoters generally results in decreased gene activity. An initial mutation dataset was established, consisting of more than 79,278 mutated promoters with one to multiple mutation sites.

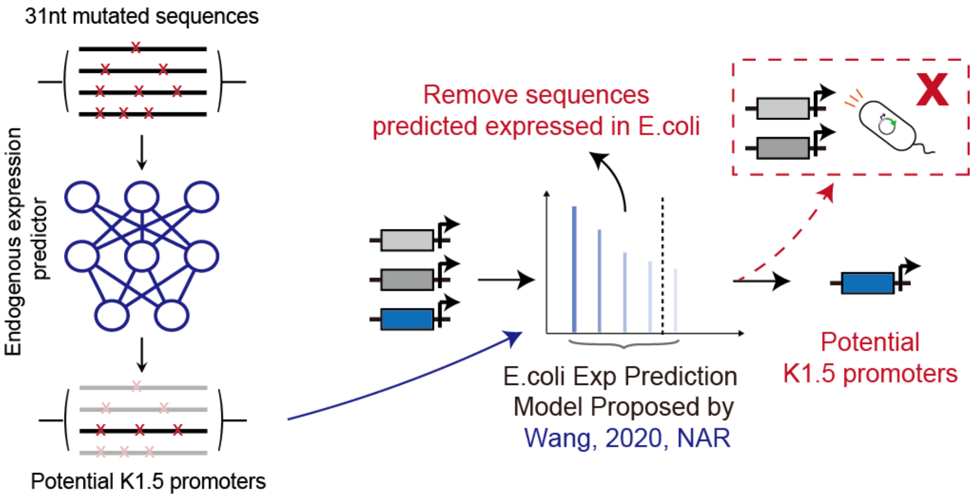

Supplementary Figure 6. Computationally Removing the E. coli Host Effect. A neural network was trained using E. coli endogenous gene expression data to predict the host expression of input sequences. The 31-nucleotide mutated sequences were input into the neural network, and sequences with high expression levels were filtered out.

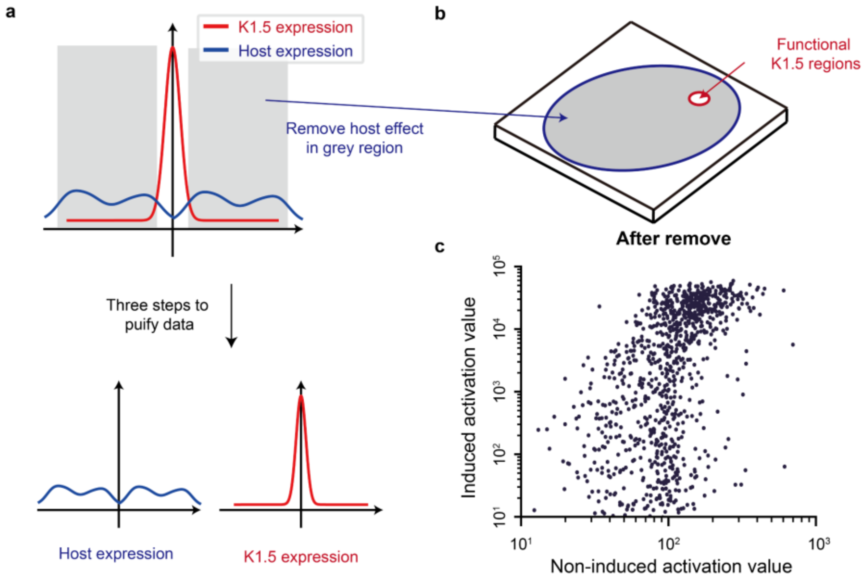

Supplementary Figure 7. Removing Leaked Host Effects. a, the observed gene activity can be divided into two components: K1.5 functional gene activity and host gene activity. b, in most regions of the sequence space, host activity contributes the majority of the observed gene activity. Therefore, the K1.5 activity can be determined by calculating the difference between the observed activity and the host activity. c, the K1.5 gene activity in the dataset, which is calculated by subtracting the host activity from the observed activity.

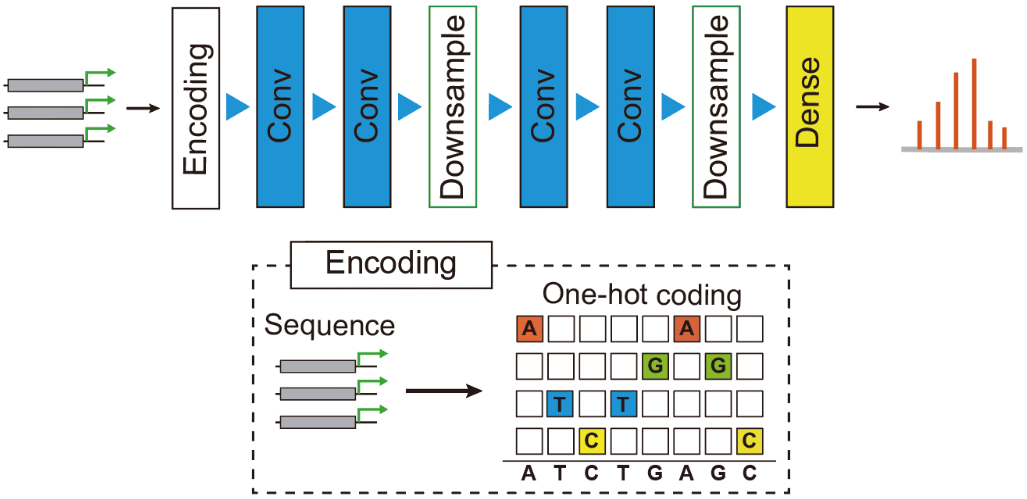

Supplementary Figure 8. The deep learning model is structured with four convolutional layers and two down sampling layers. The input sequences are encoded using one-hot encoding.

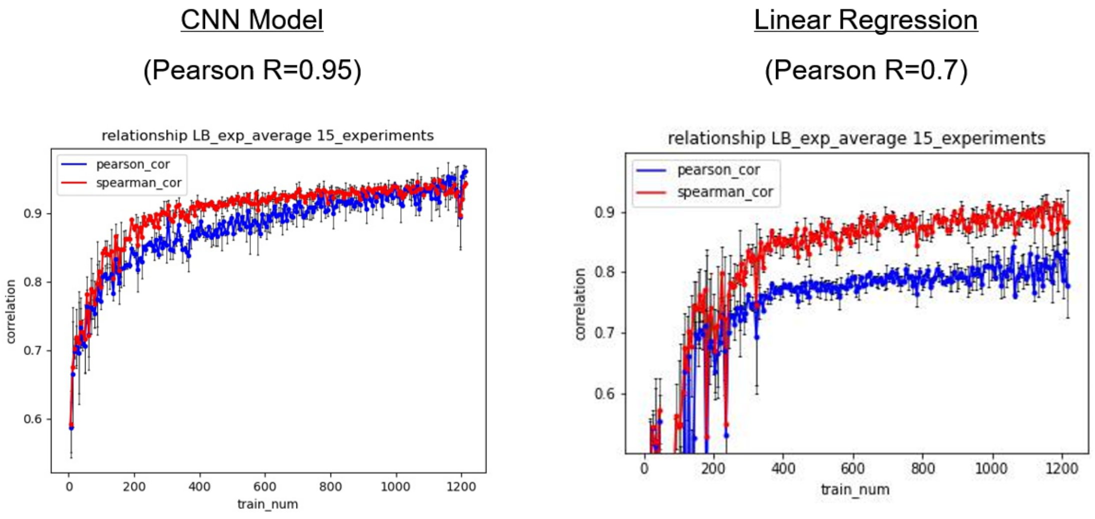

Supplementary Figure 9. The comparison of prediction results that is based on a linear regression model and a CNN model. The image illustrates how prediction results improve with an increasing number of training data. The left side shows the performance of the CNN model in our work, while the right side displays the performance of the linear regression model.

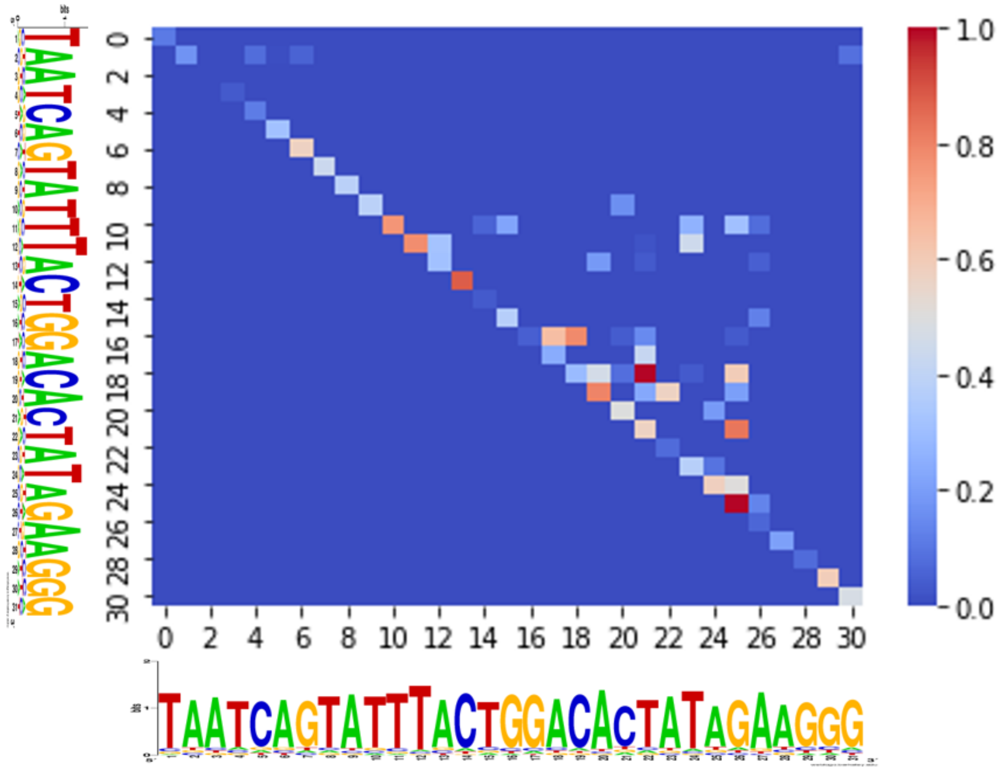

Supplementary Figure 10. A non-linear effect is present in the regulation of the K1.5 RNA polymerase gene. To demonstrate this, a second-order (k=2) polynomial model was trained using K1.5 data. The figure illustrates the strength of the non-linear effect within the model, excluding the diagonal positions.

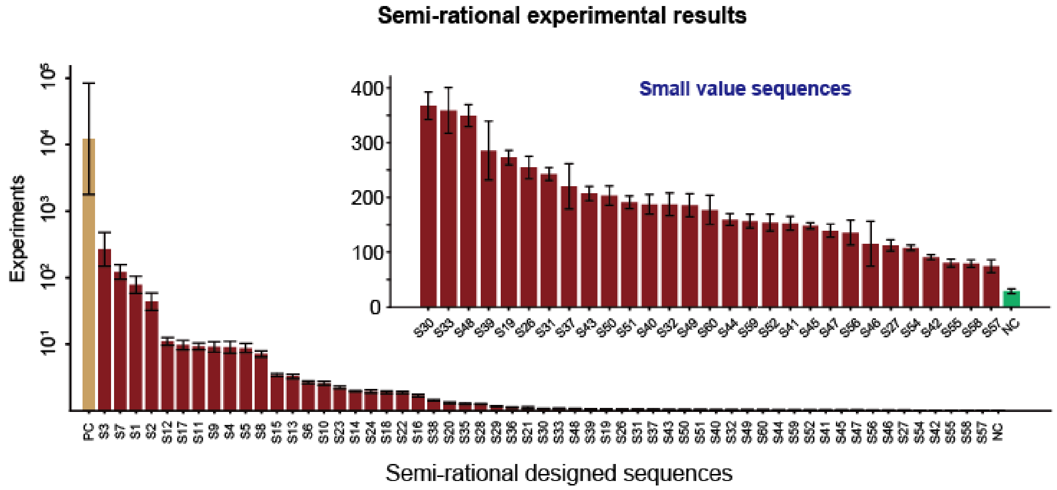

Supplementary Figure 11. The gene expression level was fixed using semi-rational design. The bottom figure represents the complete expression levels, while the top figure zooms in on the low expression region. In the figures, red indicates the designed sequences, green represents the negative control, and brown represents the positive control.

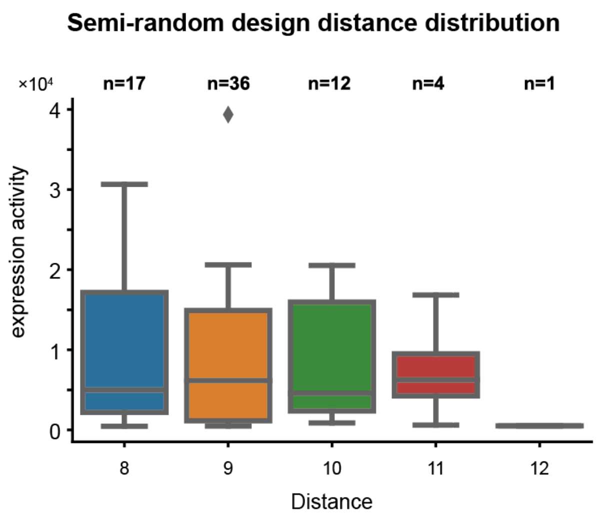

Supplementary Figure 12. The figure illustrates the distribution of gene activity as the edit distance increases in semi-rational design. It shows the edit distance between the designed sequences and the wild-type sequence. The top of the figure indicates the number of sequences at each distance, while the vertical axis represents the expression levels of these sequences

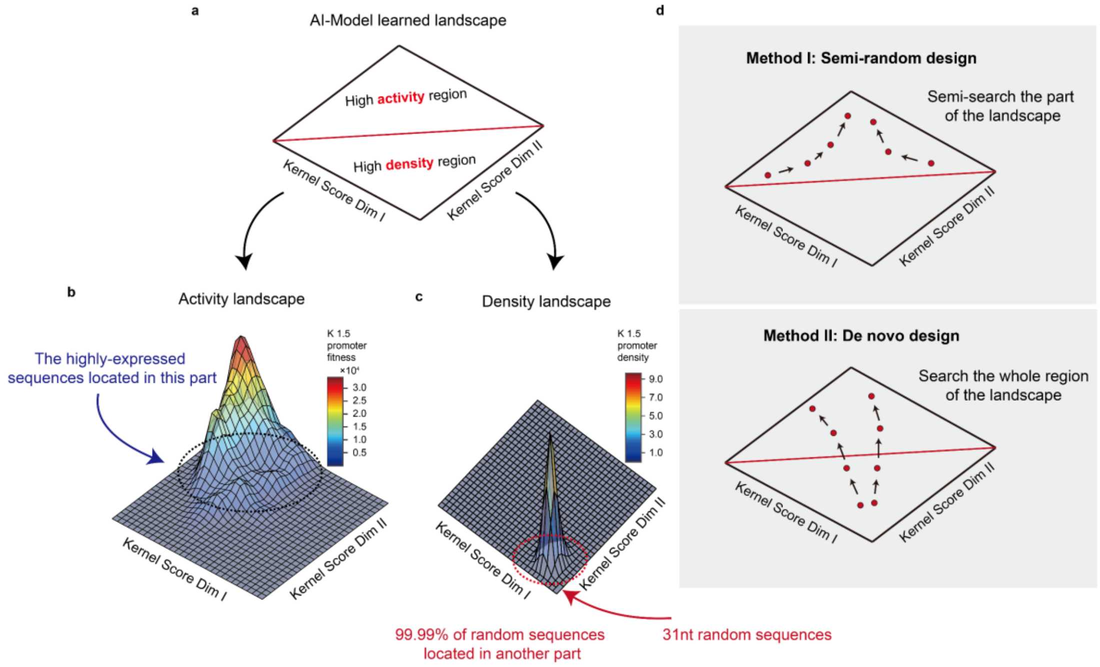

Supplementary Figure 13. The comparison between semi-rational design and *de novo* design is illustrated as follows: **a**, In the gene activity landscape, high-activity regions and high-density regions are mostly distinct from each other. **b** and **c**, The distribution of highly-expressed sequences and random sequences is depicted in the figure. **d**, Semi-rational design typically begins from the highly-expressed regions in the landscape, covering only a portion of it. In

contrast, *de novo* design generally starts from high-density regions, where 99.99% of random sequences are located, and the optimization route covers most of the landscape.

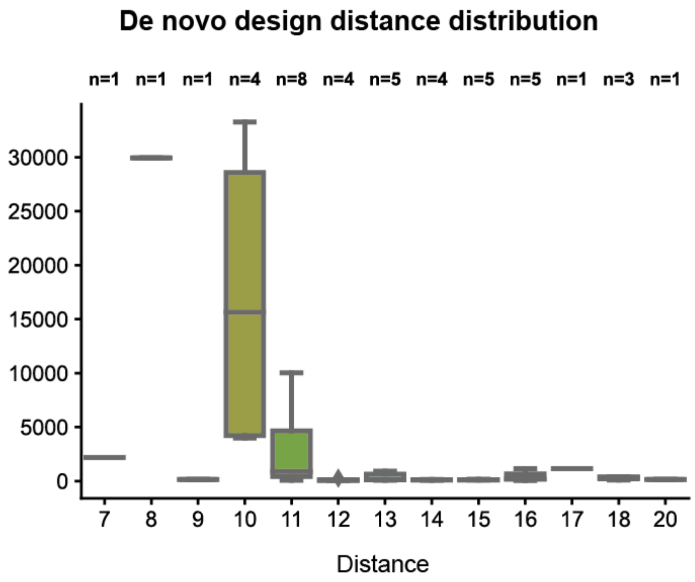

Supplementary Figure 14. The figure illustrates the distribution of gene activity as the edit distance increases in *de novo* design. It shows the edit distance between the designed sequences and the wild-type sequence. The top of the figure indicates the number of sequences at each distance, while the vertical axis represents the expression levels of these sequences

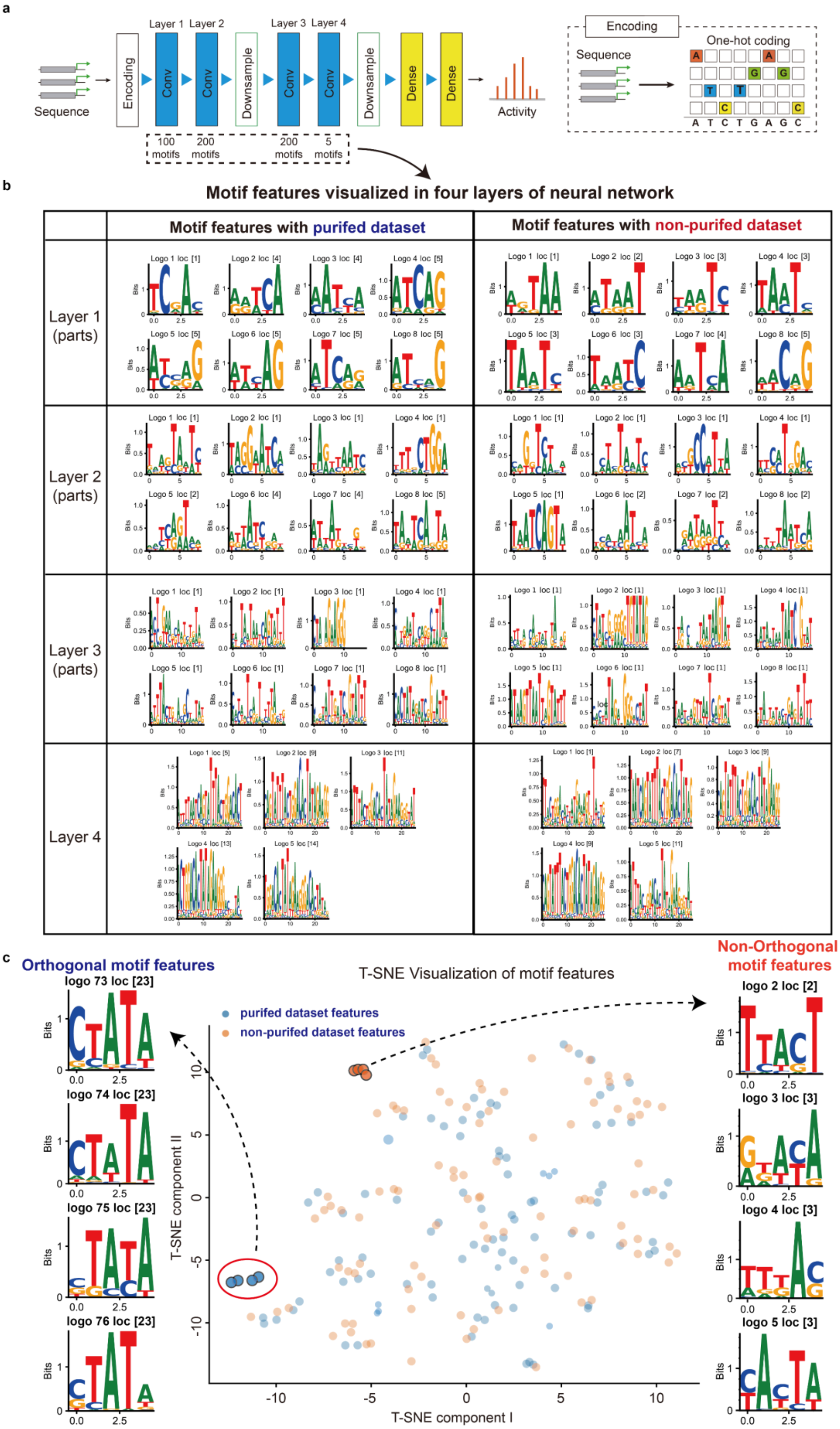
a
Sequence
Encoding
Layer 1
Layer 2
Conv
Conv
Downsample
Layer 3
Layer 4
Conv
Conv
Downsample
Dense
Dense
Activity
100 motifs
200 motifs
200 motifs
5 motifs
Encoding
Sequence
One-hot coding
A T C T G A G C
b
Motif features visualized in four layers of neural network
Motif features with purifed dataset
Motif features with non-purifed dataset
Layer 1 (parts)
Layer 2 (parts)
Layer 3 (parts)
Layer 4
c
Orthogonal motif features
logo 73 loc [23]
logo 74 loc [23]
logo 75 loc [23]
logo 76 loc [23]
T-SNE Visualization of motif features
purifed dataset features
non-purifed dataset features
T-SNE component I
T-SNE component II
Non-Orthogonal motif features
logo 2 loc [2]
logo 3 loc [3]
logo 4 loc [3]
logo 5 loc [3]

Supplementary Figure 15. The discovery of orthogonal features in the deep learning model is illustrated as follows: a, visualization methods for the deep learning model: The kernels in each layer of the deep learning model were extracted and visualized. b, motif features visualized in the four convolutional layers of the deep learning model. The left and right side showed the features trained with and without a purified dataset. "loc" represents the position in the original sequence where the motif most frequently appears, indicating the sequence location signal that the motif is most likely to capture. c, T-SNE visualization of the first layer's motif features, highlighting the differential features between the deep learning models trained with and without the purified dataset, which are considered orthogonal features.

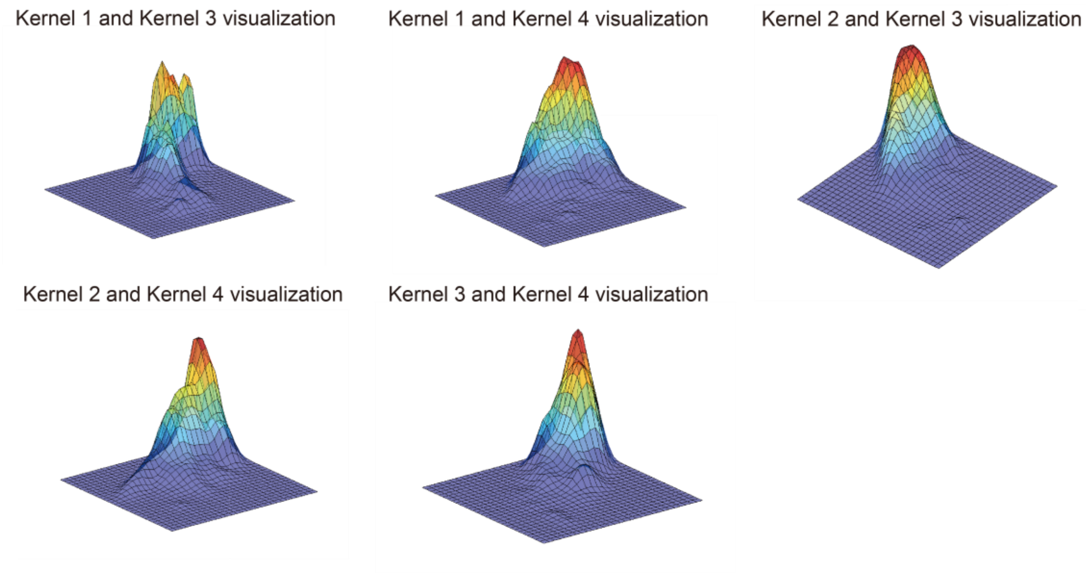

Supplementary Figure 16. The Visualization results of all kernels in the last layer. We plotted the visualization results for the combinations of kernels other than those provided in the Fig. 5. The Kernel 5 serves as an auxiliary without specific determined patterns; therefore, we have not included its visualization results in the plot.

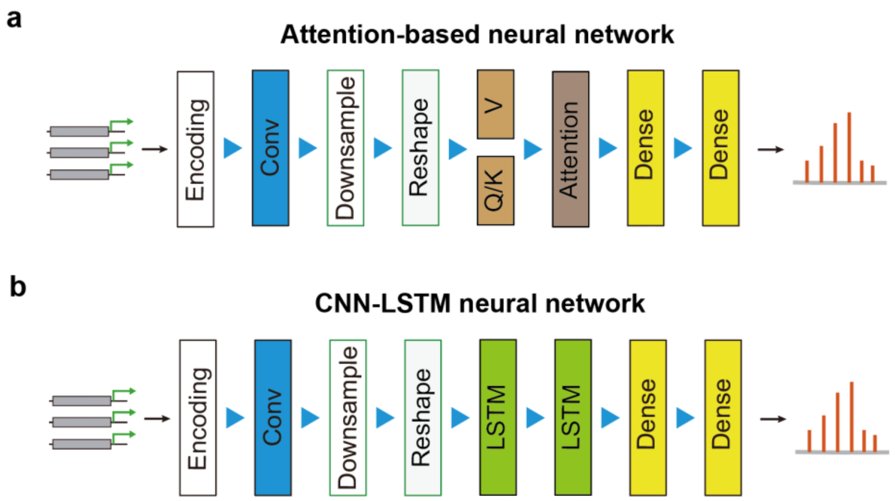

Supplementary Figure 17. The neural network structure of Attention-based neural network and CNN-LSTM neural network.

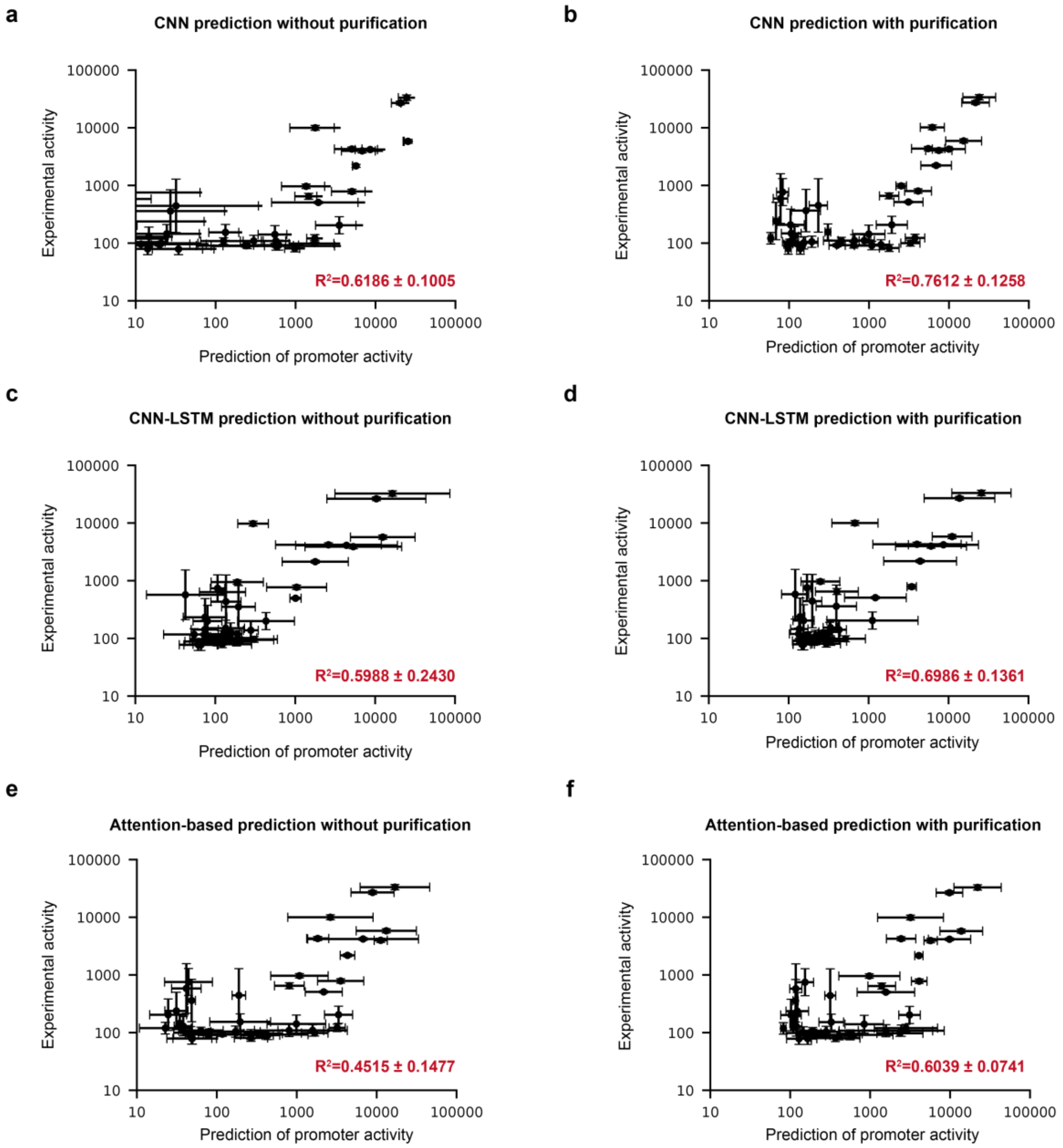

Supplementary Figure 18. Comparison of model performance across three replicates for CNN, CNN-LSTM and attention-based architectures, with and without purification. Detailed results are provided in Table S7.
